# Building capacity for water, sanitation, and hygiene programming: Training evaluation theory applied to CLTS management training in Kenya

**DOI:** 10.1016/j.socscimed.2016.08.008

**Published:** 2016-10

**Authors:** Jonny Crocker, Katherine F. Shields, Vidya Venkataramanan, Darren Saywell, Jamie Bartram

**Affiliations:** a The Water Institute at UNC, Department of Environmental Sciences and Engineering, University of North Carolina at Chapel Hill, CB #7431, 135 Dauer Drive, Chapel Hill, NC 27599-7431, USA; b Plan International USA, 1255 23rd Street NW, Suite 300, Washington, DC 20037, USA

**Keywords:** Kenya, Water, Sanitation, CLTS, Training evaluation, Capacity building, Conceptual framework, Sustainable development goals

## Abstract

Training and capacity building are long established critical components of global water, sanitation, and hygiene (WaSH) policies, strategies, and programs. Expanding capacity building support for WaSH in developing countries is one of the targets of the Sustainable Development Goals. There are many training evaluation methods and tools available. However, training evaluations in WaSH have been infrequent, have often not utilized these methods and tools, and have lacked rigor. We developed a conceptual framework for evaluating training in WaSH by reviewing and adapting concepts from literature. Our framework includes three target outcomes: learning, individual performance, and improved programming; and two sets of influences: trainee and context factors. We applied the framework to evaluate a seven-month community-led total sanitation (CLTS) management training program delivered to 42 government officials in Kenya from September 2013 to May 2014. Trainees were given a pre-training questionnaire and were interviewed at two weeks and seven months after initial training. We qualitatively analyzed the data using our conceptual framework. The training program resulted in trainees learning the CLTS process and new skills, and improving their individual performance through application of advocacy, partnership, and supervision soft skills. The link from trainees' performance to improved programming was constrained by resource limitations and pre-existing rigidity of trainees’ organizations. Training-over-time enhanced outcomes and enabled trainees to overcome constraints in their work. Training in soft skills is relevant to managing public health programs beyond WaSH. We make recommendations on how training programs can be targeted and adapted to improve outcomes. Our conceptual framework can be used as a tool both for planning and evaluating training programs in WaSH.

## Highlights

• We present a framework for planning and evaluating training in water and sanitation.
• The training led to learning and improved individual performance among trainees.
• Programming outcomes were constrained by resource limitations and rigid policies.
• Training in WaSH can be targeted and adapted for enhanced outcomes and impacts.

## 1 Introduction

Globally 2.4 billion people lack access to improved sanitation, and 946 million lack access to any sanitation facility and practice open defecation (OD) (WHO/UNICEF, 2015). Poor sanitation and hygiene together cause an estimated 577,000 deaths annually (Prüss-Ustün et al., 2014), and half of child stunting can be explained by OD (Spears, 2013). Sanitation can lead to improved social status and dignity (Jenkins and Curtis, 2005, Jenkins and Scott, 2007), gender-equity benefits (Mahon and Fernandes, 2010), and increased school attendance for girls. Human resource development has been recognized as critical to global water, sanitation, and hygiene (WaSH) progress since 1982 (Carefoot and Gibson, 1984, WHO, 1982). A 2014 global assessment found only one-third of countries had human resource strategies for WaSH, despite a lack of capacity constraining the sector (WHO, 2014). The capacity gap in WaSH includes a lack of soft skills among program managers such as partnership and supervision (WHO, 2010), which are increasingly important given a shift in WaSH interventions towards participatory behavior-change approaches that necessitate these skills (Evans et al., 2014). This gap is partly due to training that is not matched to needs, and to ill-equipped training institutions (Cavill et al., 2011). Responsibility for WaSH programs is frequently decentralized to local government without sufficient staff and financial resources (IWA, 2013). Training in soft skills has the potential to benefit public health programs beyond WaSH, by improving program planning (Pappaioanou et al., 2003), and strengthening health systems (Rowe et al., 2010). With population growth and the United Nations’ adoption of the Sustainable Development Goals (SDGs), which expand national WaSH targets to include universal access and increased quality of WaSH services (UN General Assembly, 2015), the gap between human resource capacity and targets will grow. In response, SDG Target 6a is to “… expand international cooperation and capacity-building support to developing countries in water and sanitation related activities and programmes …” (UN General Assembly, 2015).

There are few training evaluations in WaSH, and those that do exist tend to lack rigor. A review of 104 WaSH organizations found that over 60% do not monitor or report on their training programs, and only 15% monitor beyond simple process indicators such as number of trainees (Ngai et al., 2013). The review also found widespread duplication of efforts, and negligible long-term trainee tracking. There are many training evaluation frameworks and tools outside the WaSH sector; however, they require adaptation and expansion for use in the complex WaSH sector, as they tend to focus on ideal trainees and isolated environments, and assess clearly defined and easily measured learning and behavior outcomes such as performance in flight simulators and building Lego models (Adams et al., 1999, Blume et al., 2009). Evaluations of training and capacity building in WaSH tend to focus on real-world settings, but few draw on the extensive evidence, tools, and theory outside WaSH. Despite a large and growing capacity shortfall, training in WaSH is insufficient and poorly evaluated. There is opportunity to increase beneficial impact by applying well-developed theory to increase learning, behavior change, and program outcomes arising from investments in training.

We reviewed and adapted training evaluation theory, developed a conceptual framework for use in evaluating training in WaSH, and applied it to a community-led total sanitation (CLTS) management training program for government officials in Kenya. CLTS is an adaptive, participatory approach, and managing CLTS requires a diverse set of skills and collaboration between sectors. This provided an opportunity to explore the value and relevance of our conceptual framework. Our study provides new tools for use in the WaSH sector, as well as new evidence on building capacity within local government for managing WaSH programs.

## 2 Methods

This study involved the development of a conceptual framework for evaluating training in WaSH, a CLTS management training program delivered by Plan International Kenya (Plan) to government officials in Kenya, and an evaluation of the training program in Kenya using the conceptual framework.

### 2.1 Context

CLTS emerged in the year 2000 as a participatory approach to address OD (Kar and Chambers, 2008), and is now a well-established approach that has been implemented in over 50 countries (IDS, 2011). CLTS was introduced to Kenya in 2007. At the inception of this project in 2011, CLTS in Kenya was focused on policies, strategies, and institutional arrangements nationally; and on village-level implementation locally (Crocker and Rowe, 2015). The government adopted CLTS into national sanitation policy and published CLTS guidelines (Government of Kenya et al. (2011); Kenya Ministry of Public Health and Sanitation (2011)). The Ministry of Public Health and Sanitation (MOPHS) was tasked with managing CLTS programs (Crocker and Rowe, 2015). The government initiated an *Open Defecation Free* (ODF) *Rural Kenya 2013* campaign, and contracted a non-governmental organization (NGO), the Kenya Water and Health Organization, to independently verify ODF communities.

In 2013, a new constitution was enacted, in which government decision-making was devolved to 47 newly designated counties comprising 290 sub-counties. County and sub-county responsibilities are still developing and some confusion persists. The MOPHS was merged with the Ministry of Health (MoH), while other ministries are being combined or phased out.

Multilaterals and NGOs such as UNICEF, Plan, and World Vision support CLTS in Kenya financially and through training and guidance. Public health officers (employees of the MoH) and volunteer community health workers facilitate CLTS activities. However, training local government officials to manage CLTS programs, including coordinating a diverse range of organizations, has largely been overlooked (Crocker and Rowe, 2015). A lack of local government capacity to manage programs can lead to a lack of support and guidance to communities.

### 2.2 Program description

Plan identified local government (the “sub-county” in Kenya) as critical to improving CLTS programs, due to its roles in advocating to county government for policies and funding, and coordinating implementation by field officers and NGOs. Plan developed a CLTS management training program (Fox et al., 2013), and invited officials with a direct or indirect role in sanitation to participate. Plan trained officials from two sub-counties in Kilifi County, and two in Homa Bay County.

The training program comprised an initial five-day training, and “training-over-time” activities over the following seven months, which is less common than one-time training (Baldwin et al., 2009). Officials were trained by county. The initial training covered CLTS implementation with a field demonstration, and management skills in partnership, supervision, resource mobilization, and monitoring. Training was participatory, inter-ministerial, and included group work. Training-over-time activities incorporated training and application, and included CLTS field training, resource mobilization, work planning, monitoring, advocacy, training division-level staff, and sensitizing county officials. A majority of trainees attended the majority of activities. Advocacy training and sensitizing county officials were not completed in Kilifi County due to government training for polio vaccination and terrorism response taking priority. A timeline of activities with the number of trainees at each is in Supplement 1. A description of the training program is available online (Plan International Kenya, 2015).

### 2.3 Study design

We used a qualitative study design to evaluate the CLTS management training program, and developed a conceptual framework to guide the evaluation. The conceptual framework is presented in section 3.

#### 2.3.1 Data collection

**Fig. 1:**
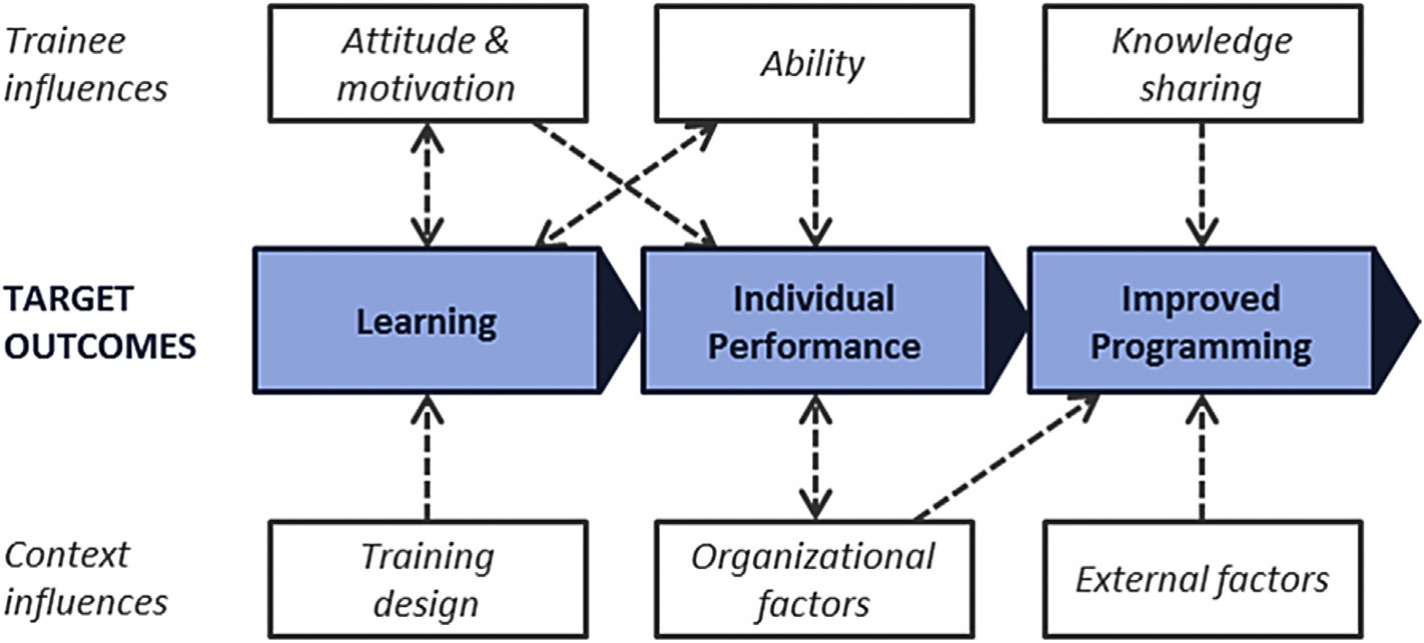
**Conceptual framework for evaluating training programs in WaSH**. This framework is an adaptation of concepts from training evaluation literature for practical use in WaSH studies.

Data collection tools (Supplement 3) were designed to identify the target outcomes of the training program (Fox et al., 2013), influences, and the links between them, following our conceptual framework (Fig. 1). Research Guide Africa (RGA), a Kenyan social research agency, was hired to recruit study participants and administer surveys and interviews. During interviewer training, the tools were tested and refined for clarity and cultural appropriateness. Four local interviewers were trained for two days, and two were hired based on performance during training. The researchers' had no interactions with trainees. All government officials who participated in the initial training were eligible for inclusion in the study. RGA obtained informed consent in person before training began. IRB approval was obtained from the University of North Carolina and from the Kenya National Council for Science and Technology. There were three interactions with trainees: 1) a pre-training questionnaire to understand trainees' background and expectations; 2) round-one interviews two weeks after initial training to assess learning, attitudes, motivations, ability, and the quality of the training design; and 3) round-two interviews seven months later after training-over-time activities to assess trainee's performance, and organizational and external factors that influenced their performance.

Individual in-depth interviews were conducted in trainees’ workplaces for 20–75 min. Interviews were in English with explanation in Swahili when necessary. Interviews were recorded and transcribed verbatim by the interviewer, with Swahili translated into English, then checked in full by the second interviewer. During round-one, interviewers did not always have time for the last few questions, so the round-two interview guide included more instructions, prompts, and timing guidance.

#### 2.3.2 Analysis

Interview transcripts were coded using Atlas.ti by the second author, and a sample was reviewed by the first author. Transcripts were coded first by interview section, then by inductive and deductive themes. The second author did not contribute to the conceptual framework until after coding to reduce the potential for bias during inductive coding. Themes were systematically categorized into outcomes and influences from the conceptual framework through discussion between the first and second authors until consensus was achieved (Fig. 1), then the framework was used to interpret themes and understand links between outcomes and influences. Selected interview quotes appear in-text with additional quotes in Supplement 4.

Knowledge gained was assessed by looking for training concepts recalled during interviews. Skills are not easily measured by interviewing, so *potential* skills were assessed from round-one interviews by asking trainees to describe how and why they planned to change their work practices. Changes made to work practices were assessed from round-two interviews administered seven months later. To avoid positive bias, trainees were prompted for detailed examples of planned or actual changes to work practices, and why they made these changes. For the purposes of analysis, general descriptions and examples—when trainees could not provide details on the how and why—were considered a lack of evidence. Factors that influenced trainees and training outcomes were assessed in two ways: by asking trainees about them directly, and by coding influencing factors when they arose naturally. Findings are not intended to comprehensively describe training outcomes and influences; rather, the most important outcomes and critical influences are revealed by this analysis.

## 3 Conceptual framework

We reviewed the literature for training evaluation frameworks, tools, and concepts, and combined and adapted them to develop a conceptual framework as a tool for identifying outcome, trainee, and context indicators, and to relate training to outcomes (Fig. 1). We also assessed the relationship between influences and outcomes in order to make recommendations for adapting future training programs to local context.

### 3.1 Literature review

We first searched for any published articles that evaluated training or capacity building in WaSH, and that used any framework, model, or guideline for the evaluation. We were looking specifically for concepts applicable to training evaluation, so included capacity building and development in our search as training is often a component of these. We found five articles that evaluated training in WaSH. Two of the studies did not use a framework or follow an evaluation guideline or protocol (Barat et al., 2014, Gunawardana et al., 2013). Two others mentioned frameworks, though they did not thoroughly describe their use (Mvulirwenande et al., 2013, Ngai et al., 2014). The fifth study proposed an approach to evaluating capacity development partnerships (Pascual Sanz et al., 2013), but was not well-suited for training evaluation. None of these five studies described analysis, and only one included their survey guides and made the link between data and results explicit (Gunawardana et al., 2013).

We did not use any of these five articles to inform our conceptual framework. We turned to training evaluation literature from outside WaSH to develop our conceptual framework. We searched for any published articles with an explicit focus on training evaluation or transfer of training. In order to cover many different evaluation approaches we only reviewed articles that present new frameworks or concepts and reviews. We began with the most cited and earliest published articles, then continued to review articles until we reached saturation (i.e. reviewing additional articles did not yield new concepts). We reviewed a total of 30 articles. The full literature review and complete definitions from our conceptual framework are presented in Supplement 2.

### 3.2 Conceptual framework

WaSH training programs are implemented to improve management and implementation of programs that construct infrastructure, deliver WaSH services, or target behavior change. Our conceptual framework includes three categories of “target outcomes,” which relate to the objectives of the organization leading the training program, and six categories of “influences,” which are factors that affect outcomes. We use the term *target* to convey that training programs should be evaluated against the target outcomes (objectives) of the organization leading the training, and that other outcomes of training may occur. This also requires that the organization leading the training sets objectives in advance. The broad outcome and influence categories and their interactions are presented in Fig. 1. Definitions of each category, including sub-categories or constructs, are presented in Table 1. Additional explanation of the conceptual framework and commentary on measurement of each outcome and influence are presented in Supplement 2.

We include three target outcomes of training: learning, individual performance, and improved programming. Improved programming leads to impacts, which are not included in the framework, because they may occur long after training ends, and the causal link is confounded by many factors that cannot be measured or accounted for (Schmidt, 2014).

We include six influences in the framework: attitude and motivation, ability, knowledge sharing, training design, organizational factors, and external factors. The first three are “trainee influences” (characteristics of trainees), and the last three are “context influences” (characteristics of the training program and work environment).

## 4 Results

### 4.1 Trainee characteristics

All 42 eligible trainees enrolled. One trainee each from Homa Bay and Kilifi Counties did not participate in the second interview. Table 2 presents trainee characteristics.

### 4.2 Evaluation findings: learning outcomes and influences

**Fig. 2:**
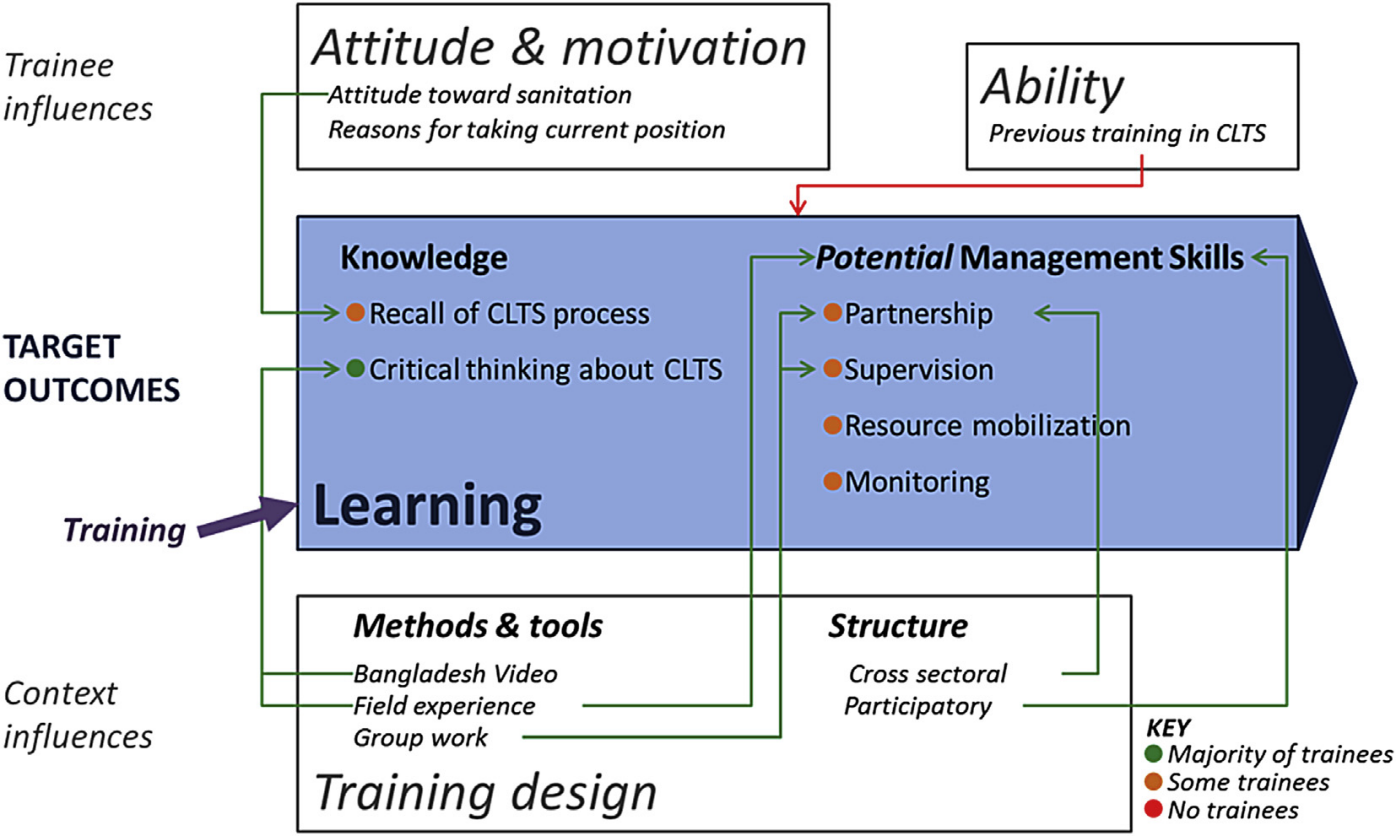
**Findings on learning outcomes and influences from evaluating a training program in Kenya, organized by the conceptual framework**. Broad categories (learning, attitudes and motivation, ability, and training design) are from the conceptual framework. Text within these broad categories are selected findings from the Kenya evaluation. Red arrows indicate a negative influence, and green indicate a positive influence.

Learning outcomes and influences were assessed from round-one interviews, so pertain only to initial training. Learning from training-over-time activities is revealed through changes in individual performance (section 4.3). Target learning outcomes of the training program were understanding the CLTS process, critical thinking around CLTS, and development of four management skills: partnership, supervision, resource mobilization, and monitoring (Fig. 2).

#### 4.2.1 Learning outcomes

Trainees were taught the CLTS steps (pre-triggering, triggering, follow-up, verification, and celebration) during initial training, and participated in triggering a community. In round-one interviews, nearly all trainees recalled the triggering that they had seen in the field, and half gave detailed examples of triggering activities. Only a quarter of the trainees recalled details on pre-triggering and follow-up. Recall of triggering details may have been higher because trainees had seen these activities in practice. No trainees gave detailed descriptions of CLTS verification or celebration. While trainees’ descriptions of CLTS triggering indicate understanding of the theory of CLTS and the activity they observed in the field, comprehensive recall of the entire CLTS process was found to be low.

During initial training, favorable and challenging conditions for implementing CLTS were presented to trainees (Fox et al., 2013). From these, trainees frequently recalled environmental and geographic conditions; however, government structure and responsibility were infrequently mentioned in interviews. Some trainees described conditions that were not covered in training, indicating trainees were thinking critically about what they had learned. For example, they described how human and financial resources, culture, and socioeconomic status could affect CLTS success, which they had not been explicitly taught. Trainees also observed conditions favorable for CLTS during the field visit: “there is need to work closely with the Provincial Administration, the opinion leaders, and also walking there earlier and walking around physically in the area, to see if that problem (OD) exists, and of course familiarizing yourself with the area.” Through recognition of these conditions, trainees demonstrated their understanding of the importance of context for success of CLTS programs. Complete trainee-identified success and challenge factors are in Supplement 5.

*Potential* skills learned were seen when trainees planned to apply skills to their work—which is a proxy for skills learned. While some trainee responses suggested learning of skills (n = 15/42), the majority of trainees could not articulate how they planned to apply new skills to their work practices. Supervision and partnership plans included forming an inter-ministerial committee to coordinate supervision of field staff (n = 1/42) and creating a forum for CLTS coordination (n = 3/42). Resource mobilization plans included approaching a new funding source (n = 1/42), and increasing follow-up and face-time with funders after submitting a proposal (n = 1/42). Monitoring plans included moving to consistent indicators across ministries (n = 1/42), and adding new indicators such as water committee feedback (n = 1/42). Two trainees said they would just continue to ask Plan for support, indicating they had not gained new resource mobilization skills.

#### 4.2.2 Influence on learning: attitude and motivation

What motivated trainees in their work was complex and varied in round-one interviews, ranging from helping people (n = 15/42) and seeing changes such as health improvements in communities (n = 6/42), to seeing broad environmental, societal, or economic change (n = 8/42). Some trainees mentioned being motivated by interacting with others (n = 9/42). Delayed funding or lack of resources was commonly cited as discouraging (n = 21/42), as was feeling that their work was hectic, stressful, or overwhelming (n = 5/42).

Some trainees revealed discomfort talking about sanitation and feces. A few trainees were embarrassed when describing CLTS:

“[The] triggering process is when the community has now realized that this thing is bad for them … they draw their community and show the houses and where they live and put where they do the thing … (laughter).”

#### 4.2.3 Influence on learning: ability

Twenty-one trainees either indicated in the pre-training questionnaire (Supplement 3) that they had prior training in CLTS (n = 13/42), or were likely to have prior training given their position and level (n = 8/42) (Table 2). Prior CLTS training was more common among trainees from the MoH than from other ministries. Trainees who already possessed some CLTS knowledge and skills had less potential to learn from training. This was most evident for trainees from the MoH, many of whom perceived the highest value of the training program to be bringing staff from other ministries into a CLTS training, whereas those outside the MoH (with no prior CLTS exposure) saw value in learning the CLTS content itself.

#### 4.2.4 Influences on learning: training design

Most trainees spoke positively about initial training. Trainees liked the participatory structure (n = 10/42) and inter-ministerial group work, which allowed knowledge sharing across different sectors. One trainee explained that the sitting arrangement allowed different levels of staff to work together: “we were like in the same wave length and we could interact freely and share freely.” The frequent group work allowed trainees to practice their partnership skills.

Many trainees remembered a video about CLTS from Bangladesh (n = 19/42), describing how it helped them think about CLTS principles such as community engagement and use of local resources. Trainees frequently cited the field activity as an opportunity to gain practical knowledge (n = 7/42). Seeing training concepts applied in the Bangladesh video and in the field in Kenya helped trainees to think critically about how CLTS could work in their counties. Higher recall of the CLTS steps that were demonstrated in the field suggests the importance of practical experience for increasing target learning outcomes.

Negative feedback on initial training primarily concerned duration. Some trainees thought the initial training should have been longer (n = 11/42), whereas a few thought it was too long (n = 3/42). Some trainees expressed a wish for higher daily cash allowances (n = 7/42).

### 4.3 Evaluation findings: individual performance outcomes and influences

**Fig. 3:**
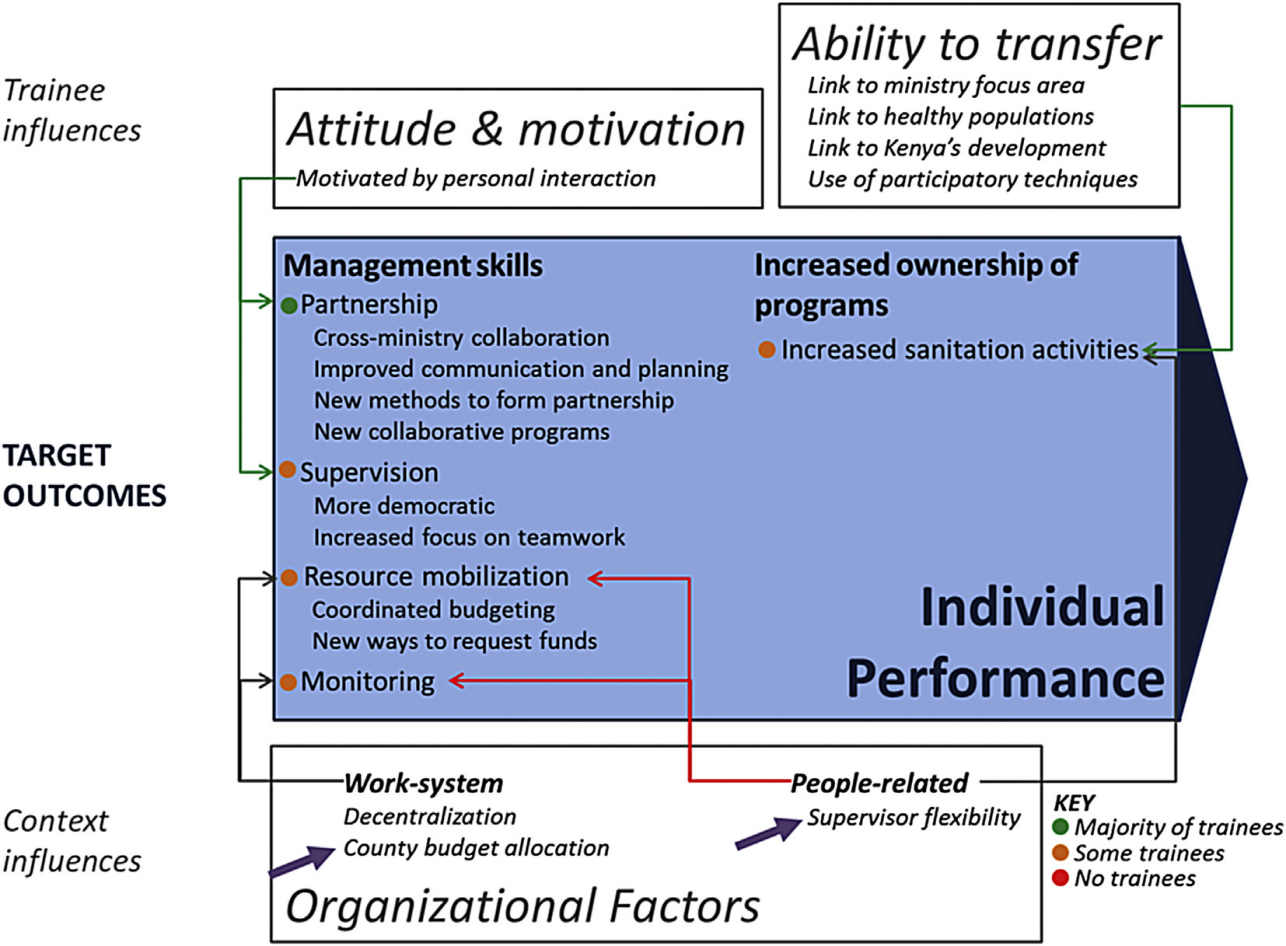
**Findings on individual performance outcomes and influences from evaluating a training program in Kenya, organized by the conceptual framework**. Broad categories (individual performance, attitudes and motivation, ability, and organizational factors) are from the conceptual framework. Red arrows indicate a negative influence, green a positive influence, and gray both positive and negative influences. Purple arrows indicate where training activities modified organizational factors.

The target individual performance outcomes of the training program were application of the four management skills to work activities, and increased ownership of sanitation programs (Fig. 3).

#### 4.3.1 Individual performance outcomes

The most commonly reported changes to work practices concerned partnership (n = 21/40) – a skill trainees practiced during inter-ministerial group work in initial training. When asked about partnerships in round-one interviews, trainees tended to list NGOs, while inter-ministerial partnerships featured prominently in round-two interviews (n = 9/40). Inter-ministerial partnerships can lead to improved coordination of CLTS programs. Trainees also described improved communication and planning with partners (n = 6/40), new methods of forming partnerships (n = 2/40), and increased collaboration on programs, such as tree planting campaigns and Global Handwashing Day (n = 2/40). One trainee noted:

“[the training program] bonded us so much that nowadays when you are calling colleagues for an activity, these are people that you are already working with and you are comfortable with them. Recently, a new NGO was coming in to collect baseline data, I simply cross over to [name] here at water office [who] immediately knows what kind of information I need.”

After initial training, trainees discussed plans for an inter-ministerial committee to supervise field staff and appraise each other's work. In round-two interviews, trainees reported they had improved communication and engagement with their supervisees in decision making (n = 4/40). One trainee reported learning new conflict resolution strategies from training.

Several trainees recalled training in resource mobilization and commented on how helpful it was (n = 7/40). Trainees experienced with CLTS made connections between resource mobilization and partnership, speaking about joint-budget planning with county government and other ministries (n = 2/40). Those without CLTS experience gave examples unrelated to CLTS, such as writing their first proposal (n = 1/40), and using new mechanisms to request funding (n = 4/40). No one mentioned mobilizing resources from Plan in round-two interviews, despite having planned to. This shift indicates that trainees were thinking about resource mobilization beyond their established mechanisms.

In round-two interviews, three trainees reported monitoring with increased frequency.

Increased ownership of sanitation programs manifested as trainees identifying ways they had, or planned to, apply CLTS knowledge in their work (n = 10/40). Some suggested they would spread CLTS and sanitation messages while visiting communities (n = 5/40). Others added sanitation activities to their work (n = 5/40), for example by including public toilets in a funded irrigation plan. One trainee suggested they would monitor OD while visiting communities for other projects.

Trainees also showed other signs of taking ownership of CLTS, like drafting a sanitation policy to secure long-term institutional support for CLTS. A quote from one trainee demonstrated their ownership of CLTS:

“[it] always worries me if a project ends, what will then drive the community, and there now you will need the service of the health promotion officer. Everybody will leave. Every other department will say ‘that Plan thing came to an end and we are waiting for it if it comes back! But now as a health promotion officer it is my burden to see that this continues, enablement of people's health issues continues, so what has been started, it has to move on! … if I pack up and say that ‘Plan mentorship went! EGPAF went! UNICEF went!’ Then I will be killing the community!”

The increased coordination and collaboration between ministries described above was another indication of increased ownership of CLTS.

#### 4.3.2 Influences on individual performance: attitude and motivation

The attitudes and motivations that influence learning also influence individual performance.

#### 4.3.3 Influences on individual performance: ability

Following our conceptual framework, we looked for ways in which trainees were able to make connections between training and their work (n = 18/40), which can lead to improved individual performance. Trainees saw connections in four areas: links to their ministry's focus area (n = 8/40), creating healthy populations (n = 8/40), supporting Kenya's development (n = 2/40), and applicability of the CLTS approach to their work (n = 3/40).

Several trainees made ministry-specific connections. One trainee working in agriculture noted that CLTS can improve hygiene behavior, allowing food crops to be sold more widely. A high-level administrator saw a connection between reduced OD and improved security for women and girls. Three trainees in the Ministry of Water linked reduced OD to improved water quality. Some trainees (such as those in education) noted their work depends on having healthy populations, which can depend on sanitation. One trainee demonstrated the ability to link the training to their work, describing how they used their new understanding of the sanitation-health link to motivate their colleagues:

“… we work with targets in government and we sign performance contracts, so even if you assign them these [sanitation] duties, the officers will say that it’s not within their performance contracts … But when they are taken through this process, they realize that this [sanitation] problem is affecting health issues in communities. They then realize that as an officer, when people are often sick in an area, they won't be able to mobilize them for any activities, and that he too won't achieve his targets, so that connection needs to be established.”

A few noted that CLTS is good for Kenya's development, and that sanitation is recognized as a right in their constitution. Others noted they could use triggering and participatory techniques from CLTS for other behavior change programs. For those in the MoH already working on CLTS, links between training and their work were clearer.

#### 4.3.4 Influences on individual performance: organizational factors

Organizational factors can be categorized into people-related factors and work system factors. Having an inflexible supervisor was found to be an important people-related organizational constraint. Some trainees were motivated to pursue CLTS, but found it difficult, because it was not part of their core functions (n = 3/40). One commented that:

“… at times when the trainings are organized they tend to clash … my supervisor is sometimes not willing to let go because he wonders that this is not my core function and not in my job description.”

Two training activities were directed at this constraint: Plan sensitized trainees’ supervisors at the county level to the importance of sanitation and CLTS (Fig. 3), and also trained trainees in advocacy. The sensitization modified the organizational constraint by encouraging flexibility by the supervisors so that trainees could apply their learning, while advocacy training empowered trainees to argue for increased flexibility directly. These two activities only occurred in Homa Bay county.

Rigid organizational guidelines were a work system factor that constrained changes in monitoring. One trainee commented that monitoring community-level outcomes had not changed because they always followed existing guidelines.

Insufficient financial resources were a frequently referenced work system factor constraining the application of new knowledge and skills (n = 10/40). Plan's training program included two activities directed at this constraint: lobbying county government to commit 0.5% of the following year's budget to sanitation (Fig. 3), and resource mobilization training so that trainees could raise funds themselves. However, several trainees cited their lack of authority and inability to change fixed budgets as preventing them from applying their new resource mobilization skills (n = 4/40), indicating additional organizational factors constraining individual performance.

The 2013 devolution in Kenya was an important work system organizational factor, as an enabler and constraint. For one trainee, decentralized decision-making allowed better communication with supervisors who had relocated from the capital city to regional centers. For others, devolution meant lacking a supervisor for several months (n = 3/40). Uncertainty regarding renewal of field staff contracts also discouraged trainees from applying new supervision skills (n = 2/40).

### 4.4 Evaluation findings: improved programming outcomes and influences

#### 4.4.1 Improved programing outcomes

**Fig. 4:**
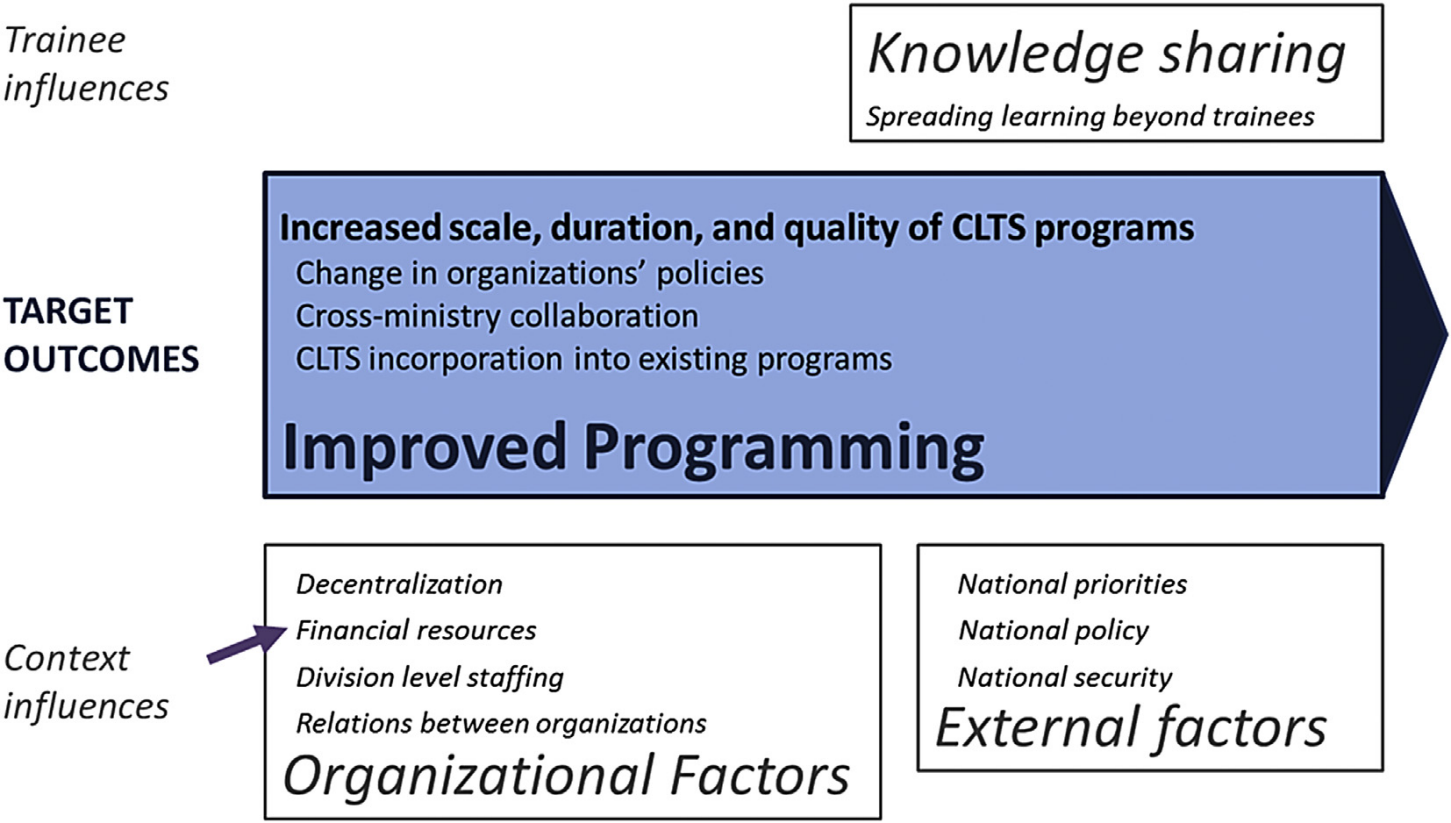
**Findings on improved programming outcomes and influences from evaluating a training program in Kenya, organized by the conceptual framework**. Broad categories (improved programming, knowledge sharing, organizational and external factors) are from the conceptual framework. Text within these broad categories are selected findings from the Kenya evaluation. Purple arrows indicate training activities.

Assessment of improved programming is often difficult and inconclusive, as outcomes can occur long after training and are influenced by many factors. Increased scale and duration of CLTS programs may only occur after our seven-month evaluation timeframe, and cannot always be linked to training when many external factors are present. We did not attempt to evaluate programming outcomes, but instead looked for preliminary indications of improved programming (Fig. 4), and asked trainees to reflect on programming. A few trainees thought the government could independently scale-up CLTS in their county (n = 10/40), though the majority thought that support from Plan or other NGO partners would be necessary. Trainees outside the MoH suggested integrating CLTS into school and agricultural programs, and youth and women's groups as mechanisms for scale-up.

#### 4.4.2 Influences on improved programming: knowledge sharing

Both interview rounds included questions on knowledge sharing—trainees taking the initiative to transfer learned knowledge and skills to their colleagues and supervisees. Many trainees reported sharing knowledge (n = 20/40), and one elaborated on its importance:

“If you don't share knowledge, it is like it is not there. So when you share knowledge, you ease the work … you cannot carry everything on your shoulders. You need to leave some of the work to others, so you delegate such that work continues even without you.”

Another trainee described how knowledge sharing can lead to training outcomes being more resilient to staffing changes: “I would like when I leave any other person who is coming in finds a system that is working, not an individual's job!” Knowledge sharing can improve programming by facilitating institutionalization and sustainability of training outcomes.

#### 4.4.3 Influences on improved programming: organizational factors

There were several work system organizational factors that influenced improved programming. Trainees from several organizations cited insufficient staffing (n = 10/40), competing responsibilities (n = 3/40) and uncertainty resulting from changing personnel and ministry restructuring during devolution (n = 16/40) as programming challenges. For example, Ministry of Education officials were unable to receive county funding for sanitation, as their ministry was not yet decentralized. Insufficient financing was described as constraining scale-up and duration of CLTS programs (n = 7/40). Plan lobbied county government directly and trained trainees in resource mobilization to address financial constraints (Fig. 4).

People-related organizational factors also influenced improved programming. Lack of trust prevented the establishment of a collective bank account for CLTS, when trainees were unable to agree on who would control the account. One trainee described tension with trainees from the MoH:

“… people have been seeing sanitation as a Ministry of Health kind of issue, so if they don't incorporate these other people who are not at the Ministry of Health and they want to go by themselves, I don't see them succeeding … all of us are targeting the community as a client and when it's all-inclusive that is the strength.”

Another described trainees from different ministries disagreeing about incorporating environmental impact assessment into CLTS. Trainees also noted that NGOs rely on government staff and expect them to drop other responsibilities to implement NGO programs (n = 2/40). While trainees were able to improve their individual performance, these organizational constraints may reduce the impact of training on improved sanitation programming.

#### 4.4.4 Influences on improved programming: external factors

External factors enable or constrain trainees and their organizations. Several trainees recognized the national policy environment as broadly enabling CLTS programs, noting that CLTS is included in the sanitation policy, and that policy can empower communities to act on their own. A few trainees described policy as constraining CLTS programs, particularly the conflict between CLTS being “community-led” and the government having a national ODF target and top-down policies. The security situation in Kilifi was an external factor that directly affected the training program, as all government officials in Kilifi were required to attend meetings on terrorism response, which delayed some training activities, and resulted in advocacy training being dropped in Kilifi.

## 5 Discussion

**Fig. 5:**
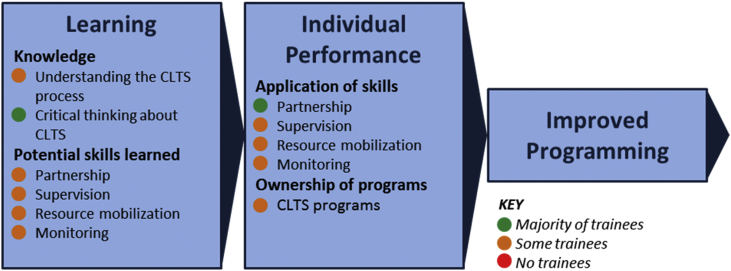
Achievement of the target outcomes of a CLTS management training program for 42 trained government officials in Kenya.

We developed a conceptual framework for evaluating training in WaSH (Fig. 1) and used it to evaluate a CLTS management training program for government officials in Kenya. The framework includes three categories of outcomes (learning, individual performance, and improved programming) which we evaluated against the training objectives (Fig. 5). The framework also sets out six categories of influences on outcomes.

### 5.1 Outcomes

The target learning outcomes of the training program in Kenya we evaluated were an understanding the CLTS process, critical thinking about CLTS, and development of management skills. After the initial training, few trainees understood the entire CLTS process, although most demonstrated critical thinking about implementing CLTS in their counties. Round-one interviews also indicated that the initial training resulted in limited learning of new skills. However, trainees later demonstrated they had gained new skills when they applied them to their work.

Target individual performance outcomes were application of management skills to work activities, and increased ownership of sanitation programs. There were frequent examples of trainees improving their work by applying new partnership skills. Improved coordination between ministries and supervision of field staff were particularly apparent. Application of other skills were less common: a few trainees had used new resource mobilization and monitoring skills. Trainees, including those with no prior CLTS experience, also demonstrated increased ownership of sanitation programs in a variety of ways such as incorporating sanitation into existing work activities.

Target improved programming outcomes were increased scale, duration, and quality of CLTS programs. No interview questions directly focused on these programming outcomes, as a longer term evaluation with comparison groups would be needed to assess them. Nevertheless, increased ownership of sanitation among trainees, and increased coordination and collaboration between ministries were indications that improved programming was likely to occur.

### 5.2 Influences on outcomes

By using our conceptual framework to guide our evaluation, we identified characteristics of trainees and the context in which they work that both constrained and enhanced the training outcomes. We discuss influences and recommendations for training together, as improving outcomes involves identifying influences in advance, then adapting training to reflect these influences (see “training needs assessment literature” for sample methodologies: Goldstein and Ford, 2002, Moore and Dutton, 1978, Rossett, 1987).

We found that a variety of aspects of the training design enhanced training outcomes. The trainers improved learning outcomes by conveying training objectives, focusing on practical knowledge and skills, and actively involved trainees, all of which are adult learning principles that should be incorporated into training of public health professionals (Bryan et al., 2009). We found that incorporation of learning-by-doing activities such as hands-on field training can positively influence trainees’ motivation, knowledge recall, and critical thinking. Videos and examples from unfamiliar settings can foster creative thinking. Group discussions and brainstorming can help trainees identify or create ways to apply their learning to their work. Participatory training emphasizing group work can improve relationships between trainees and reduce tensions between ministries. Training-over-time activities enhanced learning of new skills that were not fully developed during the initial five-day training session. Training-over-time also enhanced application of skills, consistent with non-WaSH studies which recommend unstructured, on-the-job learning opportunities as a “post-training intervention” (McCauley et al., 1994, Paradise, 2007). Training-over-time is an underutilized approach (Baldwin et al., 2009) that should be used to improve learning and performance outcomes.

A number of organizational factors negatively influenced trainees' individual performance, and constrained the link between individual performance and improved programming. A lack of flexibility on the part of trainees' supervisors, insufficient human and financial resources allocated to sanitation, slow-to-evolve relationships between organizations, and uncertain roles following devolution all initially prevented trainees from introducing new activities into their work. Organizational constraints can be addressed by empowering trainees or by having the organization delivering training modifying constraints directly. For example, we found that resource mobilization and advocacy training empowered trainees to advocate for increased flexibility and financial support. Plan also directly lobbied trainees’ supervisors (county government) for increased flexibility and budget commitments. However, direct modification of constraints does not necessarily result in increased learning or sustainable outcomes, and should be used in combination with training activities to empower trainees to address these constraints themselves. The link from individual performance to improved programming was supported by trainees sharing knowledge with colleagues, which could be encouraged as a way to reinforce learning and cost-effectively spread learning beyond trainees.

While enhancing outcomes by targeting training to favorable individuals and contexts may seem appealing, and indeed may be effective for some types of training (Crocker et al., 2016), we recommend against this strategy for management training of government officials. Managers should be trained as teams for multi-sectoral WaSH programs, and unfavorable contexts often align with the greatest need. For example, targeting only officials without prior CLTS training would have excluded MoH officials, whose presence provided trainees an opportunity to practice cross-sectoral partnership skills. Additionally, some studies have found that “overlearning” (repeating training content to embed learning) is beneficial (Burke and Hutchins, 2007).

### 5.3 Conceptual framework

The conceptual framework can be used to support the design of training programs (by implementing organizations) and their evaluation (by researchers, ideally with data collected independently of the implementing organizations to minimize bias). The three outcome categories can be used to set and organize training goals, which should be done before training begins so that they can be communicated to trainees at the outset, and so that they can be evaluated. The six influences can be assessed during a situational or needs assessment prior to training, so that the training program can be adapted to reflect these influences. Modifiable organizational constraints can be addressed in parallel to or as part of training (Baldwin et al., 2009, Eddy and Tannenbaum, 2003). To our knowledge, this is the first framework developed specifically for evaluating training in WaSH.

Our intent was to develop conceptual framework that can be used across training in the WaSH sector. The outcome and influence categories can apply universally, although the specific factors relevant within each category will vary between training programs. The tools and analysis methods used here should be adapted, modified and replaced as others feel is appropriate for different applications. For example, we focused on learning and individual performance outcomes, so only interviewed the trainees and used a seven-month evaluation period. Those wishing to focus on improved programming outcomes and external factors should consider interviewing trainees’ peers as well. While this framework is a tool to support evaluation of training, it is not a substitute for an appropriate study design, quality data collection, and analytical rigor.

### 5.4 Limitations

This evaluation had a seven-month timeframe, so long-term outcomes, such as increased scale and duration of CLTS programs, were not seen. We did not interview anyone beyond trainees. Interviews did not include questions to elucidate all influences on training outcomes. Impacts on beneficiaries’ health and wellbeing are influenced by a wide range of factors that are not all measurable and cannot be linked to training, and thus were not included in this study.

## 6 Conclusions

There is a substantive human resources capacity gap in WaSH, which will only widen with population growth and heightened service quality benchmarks and coverage targets introduced with the SDGs. In response, the need for training in WaSH will also increase. The few published training evaluations in WaSH tend to lack rigor, and do not draw on the extensive evidence that exists outside of WaSH. We reviewed training evaluation literature, developed a conceptual framework, and used it to evaluate a CLTS management training program in Kenya.

Ultimately, the training did not achieve its target outcomes among the majority of trainees. However, innovation is often the result of a few champions or opinion leaders (Rogers, 1983, Valente and Davis, 1999), so it still seems promising that there was a dramatic shift toward integration of participatory techniques and democratic management styles among several trainees, and an increased awareness of sanitation issues among a majority. Training programs for government officials should include soft skills applicable across public health sectors such as advocacy, partnership, and supervision, to increase the value of training and justify time spent away from other responsibilities.

A growing need for capacity building in WaSH combined with limited prior evaluation presents both a risk of misdirecting investments in training, and an opportunity to influence training for improved outcomes. We suggest that our conceptual framework can support design of effective training programs and more rigorous training evaluations in WaSH.

## Ethics statement

This study was reviewed and approved by the UNC Office of Human Research Ethics (study #13-0937) and by Kenya National Council for Science and Technology (reference #NCST/5/002/R/173/4). Informed consent was received from all participants.

## Figures and Tables

**Table 1:** Definitions of terms in the conceptual framework for WaSH training evaluation.

Category	Term	Definition
Outcomes	Learning	Knowledge and skills gained by trainees.
	Individual performance	Changes made to work activities by trainees through application of learning.
	Improved programming	Increased scale, duration, or quality of the outcomes of the programs in question. Increased scale refers to an increase in the number of communities or people benefiting from the programs.
Trainee influences	Attitude and motivation	Motivation to learn, and attitudes toward learning, training material, and their work.
	Ability	*As ability influences learning:* cognitive ability and prior learning. *As ability influences individual performance:* ability to transfer training content into work activities, which includes understanding the relevance of training content to their work, and ability to see opportunities to apply learning in their work.
	Knowledge sharing	Trainees passing learning on to colleagues within their organization or within partner organizations.
Context influences	Training design	Training structure (e.g. setting, sequence of training material), and methods and tools (e.g. communicating training objectives, field work).
	Organizational factors	Characteristics of trainee organizations that influence trainees' application of learning to their work activities, or that influence the links between individual performance and improved programming. These can be split into people-related and work system factors. These can include within- *and* between-organization factors, such as Memorandums of Understanding, and coordinating committees.
	External factors	Factors beyond the training program, trainees, and their organizations that influence programming.

**Table 2:** Trainee characteristics.

County	Ministry	Total	County/sub-county	CLTS experience confirmed or likely	>10 years in profession
Homa Bay	Health	8	1/7	7	3
	Education	4	0/4	2	4
	Water, Environment, Natural Resource	3	0/3	2	1
	Devolution and Planning	2	1/1	0	0
	Gender and Social Development (defunct)	2	1/1	1	1
	Interior and Coordination of National Government	2	1/1	0	0
	Labour, Social Security and Services	1	0/1	0	1
	National Treasury	1	0/1	0	1
	Youth and Sports	1	0/1	1	0
Kilifi	Health	5	1/4	4	3
	Education	1	0/1	0	0
	Water, Environment, Natural Resource	5	1/4	0	3
	Interior and Coordination of National Government	1	0/1	0	1
	Labour, Social Security and Services	1	1/0	0	0
	Youth and Sports	3	1/2	3	1
	Agriculture, Livestock and Fisheries	2	1/1	1	0
