# Supplementary material for: Building capacity for water, sanitation, and hygiene programming: Training evaluation theory applied to CLTS management training in Kenya
Source: Soc Sci Med. 2016 Oct;166:66–76. doi: 10.1016/j.socscimed.2016.08.008 (PMC5034853; doi:10.1016/j.socscimed.2016.08.008)
Supplement: Supplementary file 1 [file mmc1.docx]

**Supplement 1: timeline**

| **County** | **Activity category** | **2013** | | | | | | | | | | | | | | | | **2014** | | | | | | | | | | | | | | | | | | | | | | | |
| --- | --- | --- | --- | --- | --- | --- | --- | --- | --- | --- | --- | --- | --- | --- | --- | --- | --- | --- | --- | --- | --- | --- | --- | --- | --- | --- | --- | --- | --- | --- | --- | --- | --- | --- | --- | --- | --- | --- | --- | --- | --- |
|  |  | Sept | | | | Oct | | | | Nov | | | | Dec | | | | Jan | | | | Feb | | | | Mar | | | | Apr | | | | May | | | | Jun | | | |
| Homa Bay | Training activities |  |  |  |  |  |  |  |  |  |  |  |  |  |  |  |  |  |  |  |  |  |  |  |  |  |  |  |  |  |  |  |  |  |  |  |  |  |  |  |  |
|  | Research activities |  |  |  |  |  |  |  |  |  |  |  |  |  |  |  |  |  |  |  |  |  |  |  |  |  |  |  |  |  |  |  |  |  |  |  |  |  |  |  |  |
| Kilifi | Training activities |  |  |  |  |  |  |  |  |  |  |  |  |  |  |  |  |  |  |  |  |  |  |  |  |  |  |  |  |  |  |  |  |  |  |  |  |  |  |  |  |
|  | Research activities |  |  |  |  |  |  |  |  |  |  |  |  |  |  |  |  |  |  |  |  |  |  |  |  |  |  |  |  |  |  |  |  |  |  |  |  |  |  |  |  |

| **Activity** | **Homa Bay** | | | **Kilifi** | | |
| --- | --- | --- | --- | --- | --- | --- |
|  | **Start date** | **Duration (days)** | **Participants*** | **Start date** | **Duration (days)** | **Participants** |
| Initial training | 2013-09-16 | 5 | 24 | 2013-10-07 | 5 | 18 |
| Interdepartmental workplanning | 2013-10-01 | 1 | 13 | 2014-01-20 | 2 | 18 |
| Training DLMs on CLTS Approach | *NA* |  |  | 2014-02-24 | 4 | 18 |
| Interdepartmental workplanning | 2013-11-01 | 1 | 13 | 2014-04-14 | 2 | 17 |
| Monitoring visit to triggered villages | 2014-03-03 | 1 | 7 | 2014-03-10 | 1 | 18 |
| Monitoring visit to triggered villages | 2014-03-10 | 1 | 7 | 2014-04-07 | 1 | 12 |
| Sensitization of county assembly | 2014-03-10 | 1 | 10 | *NA* |  |  |
| Training division level facilitators | 2014-03-17 | 5 | 4 | 2014-03-10 | 4 | 6 |
| Training on resource mobilization | 2014-03-17 | 3 | 24 | 2014-06-02 | 2 | 18 |
| Training division level facilitators | 2014-03-24 | 5 | 4 | 2014-05-05 | 4 | 3 |
| Advocacy training | 2014-04-14 | 4 | 24 | *NA* |  |  |
| Questionnaire distribution | 2013-09-16 | 1 | 21 | 2013-10-07 | 1 | 14 |
| Interviewer training | 2013-10-01 | 2 | *NA* | 2013-10-01 | 2 | *NA* |
| Round-one interviews | 2013-10-07 | ** | 24 | 2013-10-28 | *** | 18 |
| Round-two interviews | 2014-05-26 | ** | 23 | 2014-05-26 | *** | 17 |
| *Participants refers the number of initial trainees present. | | | | | | |
| **22 round-one Homa Bay interviews occurred within 10 days, and two occurred on October 28th. 21 round-two Homa Bay interviews occurred within 6 days, one occurred on June 5, and one occurred on July 1. | | | | | | |
| ***17 round-one Kilifi interviews occurred within 5 days, and one occurred on November 19. All 17 round-two Kilifi interviews occurred within 11 days. | | | | | | |
