# Supplementary material for: Building capacity for water, sanitation, and hygiene programming: Training evaluation theory applied to CLTS management training in Kenya
Source: Soc Sci Med. 2016 Oct;166:66–76. doi: 10.1016/j.socscimed.2016.08.008 (PMC5034853; doi:10.1016/j.socscimed.2016.08.008)
Supplement: Supplementary file 2 [file mmc2.docx]

**Supplement 2: literature review and conceptual framework**

We reviewed training evaluation literature and developed a conceptual framework as an evaluation tool to help identify outcomes, trainee and context indicators, and to explore the wider relevance of our findings. The literature review and the resulting conceptual framework and definitions of terms follow.

**OUTCOMES**

We first reviewed the most commonly used training evaluation framework—the Kirkpatrick four-level model—which was published in 1959 and has since been expanded and updated (Kirkpatrick, 1959, 1996). The Kirkpatrick model has four sequential levels of outcomes: reaction (to training), learning (from training), behavior (of the individuals trained), and results (or organizational outcomes). Many adaptations and proposed improvements to the four-level model have been developed and applied. Two of the most popular ones critique the four levels as not being distinct (three “levels” relate to individual trainees), as not being sequential (behavior and results can occur simultaneously), and the reaction level not being an appropriate outcome (Alliger & Janak, 1989; Holton III, 1996; Tannenbaum, Cannon-Bowers, Salas, & Mathieu, 1993). The Holton model (1996), developed as an adaptation and critique of the 1959 Kirkpatrick model, dropped reactions as an outcome, and replaced “behavior” with “individual performance”, and “results” with “organizational results.” We reviewed a number of systematic reviews of training evaluations, which identify many different types of outcomes, context indicators, and measurement and evaluation techniques (Arthur, Bennett, Edens, & Bell, 2003; Kraiger, Ford, & Salas, 1993; Noe, 1986). We also reviewed training transfer literature and found a number of commonly cited frameworks, which are consistent in considering learning as the first outcome, and transfer of learning to the workplace as the subsequent outcome (Baldwin & Ford, 1988; Grossman & Salas, 2011).

We include three target outcomes in our framework: learning, individual performance, and improved programming. We use the term target outcomes to convey that other outcomes not related to the objectives of the training may occur which are not explored during evaluation. Following critiques of the Kirkpatrick model, we absorb reaction to training into the “training design”, and treat “attitude and motivation” as influences, rather than as an outcome. For measurement of each of these outcomes, we follow the guidance of Baldwin, Ford, and Blume, who concluded in their 2009 review that, regardless of the framework used, training outcomes should be compared to the original training objectives. Despite being critical to evaluation, training content and objectives have been underreported in the past (Baldwin, Ford, & Blume, 2009). Further literature review that informed our choice and definitions of outcomes follows.

***Learning***

The Holton model defines learning simply as achievement of the desired or targeted learning outcomes (Holton III, 1996). Learning outcomes are generally broken into some or all of three categories: cognitive (knowledge), skill-based, and affective (attitudinal and motivational) outcomes. Definitions and measures used for these three categories have been thoroughly reviewed by Kraiger and colleagues (Kraiger et al., 1993). Knowledge includes verbal knowledge (simple recall of training material), as well as knowledge organization and cognitive strategies (understanding and ability to reflect critically on knowledge). Knowledge outcomes are generally measured through testing recall of training concepts, standardized tests, probing interview questions, and self-reporting. Skill-based learning can be summarized as improved ability to perform tasks. “Open skills” focus on principles that can be adapted to unfamiliar situations or procedures, in contrast to “closed” skills which focus on discrete steps applied to routine tasks which always lead to the same result (Baldwin et al., 2009). Skills are generally tested through performance on standardized tasks in a structured setting (which does not work well for open skills) and behavioral observation. Affective outcomes are changes in trainees’ attitude and motivation. Attitude toward a subject includes both the direction (positive or negative) and strength. Motivation includes self-efficacy (confidence in ones abilities) and goal setting (drive to set and accomplish goals).

We define learning as the knowledge and skills trainees gain. We consider attitude and motivation as influences rather than outcomes, except where there are explicit attitudinal and motivation outcomes desired by the organization leading the training program. We propose focusing measurement on the target learning outcomes desired by the organization delivering the training, and propose using semi-structured interviews which allow inductive identification of learning. For skills relevant to water, sanitation, and hygiene (WaSH), such as participatory supervision of field staff across various projects, and coordination between governmental and NGO actors, classroom-derived indicators would not reveal if a trainee gained a skill they can apply in their work. Thus, rather than measuring skills in a structured setting, we propose skills be assessed by asking trainees how they plan to change work practices. Probing questions can be used to assess the extent to which these plans reflect new skills. This may underestimate skills learned, as planned and actual changes to work practices are subject to additional influences.

***Individual performance***

The term “individual performance” as a training outcome was proposed by Holton (1996), which he defined as “change in individual performance as a result of learning being applied on the job.” The third level in the Kirkpatrick model (1959), “behavior”, is a similar concept and is defined as using learned principles and techniques on the job. The application of learning on the job is also frequently called “transfer of training”, which had been reviewed thoroughly by Baldwin and others, and has been described as applying knowledge, skills, and attitudes gained in a training context to the job (Baldwin et al., 2009; Baldwin & Ford, 1988). Individual performance can consist of changing routine tasks linked to learning closed skills (e.g. increased accuracy and speed for an unchanging procedure), or generalizing and adapting learning to new and changing situations. The latter becomes relevant when training focuses on open skills for autonomous workers (i.e. those not constantly under supervision) (Baldwin et al., 2009; Yelon & Ford, 1999). Measurement of individual performance has focused on performance on in-classroom tests such flight simulators or speed at assembling Legos (Adams, Klowden, & Hannaford, 1999; Blume, Ford, Baldwin, & Huang, 2009) which constitutes application of close skills; and on supervisor, peer, and self-ratings using standardized measures such as Likert scales (Ford & Weissbein, 1997; Kirkpatrick, 1976), which can be used as a proxy for application of open skills. For measuring wider-reaching application of open skills (such as principles for participatory collaboration and supervision) to dynamic or new situations, qualitative and inductive methods such open-ended interviews and observation of workplace behavior are more appropriate (Mertens, 2005; Patton, 2002).

We define “individual performance” as trainees changing their work activities by applying new knowledge and skills. We propose focusing measurement on target outcomes of training, by assessing the extent to which knowledge and skills learned during training are applied to work activities after training. This should focus on open-ended interviews after training to allow exploration of adaptive application of open skills to multiple situations. Where trainees are autonomous, interviews can be administered just to trainees with probing questions to assess the extent to which individual performance has changed and the validity of trainee responses. With non-autonomous trainees, interviews can be administered to their supervisors as well.

***Improved programming***

Outcomes beyond individual performance have commonly been called results, or organizational results. These outcomes are not as thoroughly explored or evaluated as the first two outcomes, as they can be delayed, and are more complex to measure and to link to training (Aguinis & Kraiger, 2009; Kirkpatrick, 1996). Kirkpatrick defined “results” as the desired results of training, with reduced costs and increased production as examples (Kirkpatrick, 1959). Holton defined “organizational results” as the changes at the organizational level that result from changes in improved performance (Holton III, 1996). A review of the broader benefits of training found that training has focused on productivity, costs, and revenue at the organizational level, and on human capital and national economic performance for any outcomes beyond organizations (Aguinis & Kraiger, 2009). Measures of organizational results have included employee and customer satisfaction, productivity (e.g. number of sales) per employee or organization, and review of financial records (Aguinis & Kraiger, 2009). Since the ultimate goal of WaSH training is improved health and wellbeing of communities (beneficiaries), these definitions are too narrow and limited for application to WaSH, and their measure is not relevant. We replace the more common results (and organizational results) with improved programming, to imply a link to beneficiaries.

We define “improved programming” as an increased in the scale, duration, or quality of the outcomes of the programs in question. In other words, the programs that trainees are implementing reach more beneficiaries, for a longer period of time, and/or provide a higher quality service. Since improved programming is influenced by factors beyond trainees, and is three steps removed from training, identifying links from training to improved programming can be difficult, particularly during the short evaluation timeframes that are the norm in training evaluations (Baldwin et al., 2009). Measurement of improved programming should include open-ended interviews or unstructured observation to capture the many ways in which the WaSH program in question has improved. Measurement can additionally cover pre-defined outcomes when appropriate, such as improved drinking water quality when training water utility staff.

**INFLUENCES**

“Influences” are any factors that influence or affect outcomes of training, and have also been called conditions of learning or transfer. Each of our three proposed outcomes (learning, individual performance, and improved programming) can be influenced by different factors. One of the first frameworks attempting to describe the full set of influences on training outcomes comes from a 1988 review (Baldwin & Ford, 1988). Baldwin & Ford developed a framework which included three broad categories of factors that influence learning—training design, trainee characteristics, and work environment; the latter two of which also influence transfer of learning (i.e. individual performance). A subsequent review updated the 1988 Baldwin & Ford framework to reflect the prior 23 years of research, maintaining the three broad categories of influences and adding the most common and influential sub-categories from the literature (Grossman & Salas, 2011). According to Grossman & Salas, training design is made up of behavioral modeling, error management, and realistic training environments; trainee characteristics are made up of cognitive ability, self-efficacy, motivation, and perceived utility of training; and work environment is made up of transfer climate, support, opportunity to perform, and follow-up. Holton’s model (1996) includes 13 influences on training outcomes; the notable influence not contained in other models is “external events” which comprises factors external to training, trainees, and their organizations.

Our framework includes six influences: attitude and motivation, ability, knowledge sharing, training design, organizational factors, and external factors. The first three are “trainee influences” (characteristics of trainees), and the second three are “context influences” (characteristics of training and the work environment). These six influences are broadly defined so as to capture all of the influences described in the Baldwin & Ford (1988), Grossman & Salas (2011) and Holton (1996) models. The links between influences and target outcomes are shown in Figure 1. Additional literature review for each influences and our definitions follow.


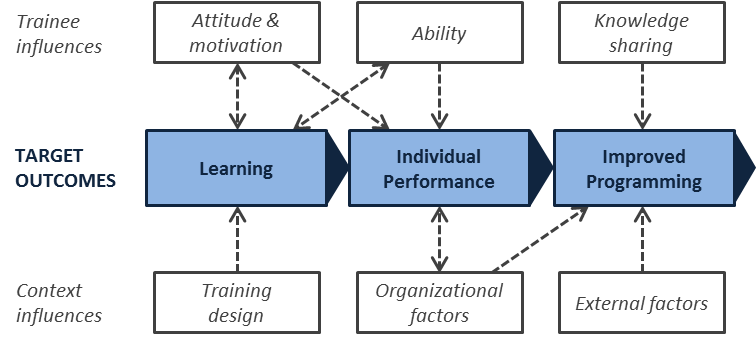


**Figure 1: Conceptual framework for evaluation of training programs in WaSH.** This framework is an adaptation of concepts from evaluation of training literature for practical use in WaSH studies.

***Attitude and motivation***

Noe (1986) reviewed trainee attributes and attitudes that influence training effectiveness. He concluded the most important trainee attributes and attitudes were “locus of control” and “self-efficacy” (which are closely linked and together reflect an individuals’ belief and confidence in their ability to influence events that affect them), attitudes toward their job and career, motivation to learn, and motivation to transfer learning to their job. Others emphasized self-efficacy and attitude towards their job as critical to training outcomes, and Baldwin additionally emphasized attitude toward the training content (Baldwin & Ford, 1988; Lim & Morris, 2006). A review proposed that one of the five adult learning principles applicable to public health practice is that “adults are motivated to learn by the need to solve problems” (Bryan, Kreuter, & Brownson, 2009). A meta-analysis of an extensive set of influences demonstrates that these same aspects of attitude and motivation also influence transfer of training (i.e. individual performance) (Blume et al., 2009).

We define “attitude and motivation” as the trainee’s motivation to learn and to improve their work, and their attitudes toward learning, training material, and their work. Attitude and motivation are preexisting in the trainee, but are dynamic and can change during or after training. We propose measurement focus on asking trainees about their attitudes and motivations related to the training content and their work, as these are areas that can be reflected in design of the training structure and content.

***Ability***

The aspects of ability that influence learning have little overlap with those that influence individual performance (unlike attitude and motivation where there is significant overlap). Ability as it influences learning has been called “trainability” (Noe, 1986) and “cognitive ability” (Holton III, 1996), and generally describes the trainees ability to both recall and understand new concepts and skills. Ability as an influence on individual performance is also a function of cognitive ability (Burke & Hutchins, 2007). However, its relevance to individual performance manifests as ability to transfer, or what Holton (1996) calls “transfer design”, essentially the extent to which trainees’ see relevance and value of training to their work and know how to apply their learning in their work. Training can include efforts to help trainees think through how to apply their learning to their work in order to enhance this aspect of ability (Holton III, 1996). A review proposed that two of the five adult learning principles applicable to public health practice relate to ability: “adults previous experience must be respected and built upon”, and “learning approaches should match adults’ background and diversity” (Bryan et al., 2009).

We define ability—as it influences learning—as cognitive ability and prior learning. We include prior learning because, for trainees who already possess some of the knowledge and skills to be taught during training, there is less they can potentially learn from training. We propose focusing measurement on assessing the trainees’ pre-training understanding of, and experience with, the training content.

We define ability—as it influences individual performance—as trainees’ ability to transfer training content into their work activities. Ability to transfer includes understanding the relevance and value of training content to their work, and their ability to see and create opportunities to apply the learning in their work. We propose focusing measurement on asking trainees how the training content is relevant to their work, and in what ways they plan to change work practices based on what they’ve learned. These questions can be followed by detailed probing to assess the depth of the trainees’ ability to transfer.

***Knowledge sharing***

Knowledge sharing – trainees sharing knowledge with colleagues of their own initiative – was explored in interviews as part of the evaluation of the management training for government officials in Kenya. We found it to be an important influence on improved programming during analysis, and added it to the conceptual framework. Knowledge sharing is not represented in evaluation of training literature, which tends to focus on trainees, though it has been referenced before in WaSH (Gunawardana, Leendertse, & Handoko, 2013).

We define “knowledge sharing” as trainees passing learning to colleagues within their organization or within partner organizations. Knowledge sharing can spread the benefits of training beyond trainees, and can help extend the duration of training outcomes by making knowledge within organizations more resilient to changes in personnel. We propose measuring knowledge sharing through open ended questions about whether trainees’ had shared knowledge from training with others. When further validation is desired, trainees’ colleagues can be interviewed to assess their gained knowledge taught during training.

***Training design***

Much research has been done on what features of training are most likely to lead to training outcomes. Baldwin & Ford’s 1988 model included three training design factors: incorporation of learning principles, sequencing of training material, and the job-relevance of the training content. Grossman & Salas’ 2011 update on Baldwin and Ford’s 1988 review added three additional training design factors: behavioral modeling (opportunities to practice new skills during training), error management (helping trainees think through workplace challenges), and a realistic training environment that resembles their workplace. A 2007 review described strategies and procedures of training design that can improve outcomes, including conducting a needs assessment, setting and communicating learning goals, overlearning (repeating training content to embed learning), and developing self-management strategies such as work plans (Burke & Hutchins, 2007). There are many guidelines for conducting a training needs assessment (for example: Goldstein and Ford, 2002; Moore and Dutton, 1978; Rossett, 1987). A 2009 review found that training-over-time activities such as helping trainees with work planning enhanced training outcomes (Baldwin et al., 2009). Others have found that the most influential training activities are often unstructured, such as on-the-job mentoring (McCauley, Ruderman, Ohlott, & Morrow, 1994; Paradise, 2007). Kirkpatrick (1959) did not specifically outline training design, but stated that trainee reactions to training were an important first step toward training outcomes. A review proposed five adult learning principles applicable to public health, two of which are directly relevant to training design: adults need to know the training objectives (i.e. why they are learning), and adults need to be actively involved in the learning process, such as through group and field work (Bryan et al., 2009).

We define “training design” as training structure (e.g. setting, sequence of training material, multiple training sessions), and methods and tools (e.g. setting and communicating training objectives, group work, field work). Rather than include specific training features in our definition, we use these two categories to capture the features of the training program in question that are found to be most beneficial or detrimental to learning. Each of the factors described in the literature fall into training structure, or methods and tools. We propose using trainees’ reactions and reflections on training to assess the relative quality of different aspects of the training program.

***Organizational factors***

Organizational factors influence both individual performance and improved programming outcomes. Organizational factors have been recognized as one of the most important influences on training outcomes (Baldwin et al., 2009; Eddy & Tannenbaum, 2003). Baldwin & Ford’s 1988 review describes organizational factors as the work environment, consisting of both support (from supervisors) and opportunity to perform (sufficient resources and flexibility to apply learning in the workplace). Other studies have shown that job description and organizational commitment to or alignment with training objectives influence training outcomes (Carlson et al., 2000; Cheng and Ho, 2001; Tannenbaum et al., 1991). More specifically, others have found that the quality of the worker-supervisor relationship (Bates, 2003), feedback and performance coaching (Mathieu, Tannenbaum and Salas, 1992; Smith-Jentsch et al., 1996; Xiao, 1996) and a group norm of openness to change (Tracey, Tannenbaum and Kavanagh, 1995) are all significant predictors of training transfer. Organizational factors can be broadly organized into two categories: people-related factors and work system factors (Diez-Roux, 1998; Lim & Morris, 2006).

We define “organizational factors” as characteristics of trainee organizations that influence trainees’ application of learning to their work activities, or that influence the links between individual performance and improved programming, which can include all of the factors listed above. We split organizational factors into the two previously mentioned categories: people-related and work system factors. Since WaSH programs often span sectors and involve multiple organizations, we expand on evaluation of training literature and include between-organization factors instead of just within-organization factors, for example interactions between organizations, and resources, policies, and strategies shared by organizations. The specific organizational factors that influence individual performance are likely to be a mix of work system and person-related factors since individual performance is at the trainee level.

***External factors***

External events have been defined as factors beyond training, trainees, and their organizations that influence programming (Holton III, 1996). This can include cultural, social, economic, political, legal, and environmental factors (LaFond, Brown, & Macintyre, 2002). There are many potential external factors that can influence outcomes beyond the trainees, and no comprehensive list can be developed (Holton III, 1996). However, training evaluations can identify what external factors are perceived to be most influential through interviews.

We use the term “external factors” and borrow Holton’s definition: factors beyond the training program, trainees, and their organizations that influence programming. We propose that assessment of external factors focus on those that most influence trainees, by interviewing them on what may enable or constrain improved programming from occurring. Broader external factors are better assessed through a situational assessment, or by referring to evaluations of the specific program in question.

**References**

Adams, R. J., Klowden, D., & Hannaford, B. (1999). Virtual training for a manual assembly task. *Haptics-E*, *2*(2), 1–7. Retrieved from http://citeseerx.ist.psu.edu/viewdoc/download?doi=10.1.1.39.6454&rep=rep1&type=pdf

Aguinis, H., & Kraiger, K. (2009). Benefits of training and development for individuals and teams, organizations, and society. *Annual Review of Psychology*, *60*, 451–74. doi:10.1146/annurev.psych.60.110707.163505

Alliger, G. M., & Janak, E. A. (1989). Kirkpatrick’s levels of training criteria: thirty years later. *Personnel Psychology*, *42*(2), 331–342. doi:10.1111/j.1744-6570.1989.tb00661.x

Arthur, W., Bennett, W., Edens, P. S., & Bell, S. T. (2003). Effectiveness of training in organizations: a meta-analysis of design and evaluation features. *Journal of Applied Psychology*, *88*(2), 234–245. doi:10.1037/0021-9010.88.2.234

Baldwin, T. T., & Ford, J. K. (1988). Transfer of training: a review and directions for future research. *Personnel Psychology*, *41*, 63–105. doi:10.1111/j.1744-6570.1988.tb00632.x

Baldwin, T. T., Ford, J. K., & Blume, B. D. (2009). Transfer of training 1988-2008: an updated review and agenda for future research. In G. P. Hodgkinson & J. K. Ford (Eds.), *International Review of Industrial and Organizational Psychology* (Vol. 24, pp. 41–71). John Wiley & Sons.

Blume, B. D., Ford, J. K., Baldwin, T. T., & Huang, J. L. (2009). Transfer of training: a meta-analytic review. *Journal of Management*, *36*(4), 1065–1105. doi:10.1177/0149206309352880

Bryan, R. L., Kreuter, M. W., & Brownson, R. C. (2009). Integrating adult learning principles into training for public health practice. *Health Promotion Practice*, *10*(4), 557–563. doi:10.1177/1524839907308117

Burke, L. A., & Hutchins, H. M. (2007). Training transfer: an integrative literature review. *Human Resource Development Review*, *6*(3), 263–296. doi:10.1177/1534484307303035

Diez-Roux, A. V. (1998). Bringing context back into epidemiology: variables and fallacies in multilevel analysis. *American Journal of Public Health*, *88*(2), 0. doi:10.2105/AJPH.88.2.216

Eddy, E. R., & Tannenbaum, S. I. (2003). Transfer in an e-learning context Francis. In E. F. Holton III & T. T. Baldwin (Eds.), *Improving learning transfer in organizations* (pp. 161–194). San Francisco: Jossey-Bass.

Ford, J. K., & Weissbein, D. A. (1997). Transfer of training: an updated review and analysis. *Performance Improvement Quarterly*, *10*(2), 22–41. Retrieved from http://onlinelibrary.wiley.com/doi/10.1111/j.1937-8327.1997.tb00047.x/abstract

Goldstein, I. L., & Ford, J. K. (2002). *Training in organizations: needs assessment, development, and evaluation* (4th ed.). Cengage Learning.

Grossman, R., & Salas, E. (2011). The transfer of training: what really matters. *International Journal of Training and Development*, *15*(2), 103–120. doi:10.1111/j.1468-2419.2011.00373.x

Gunawardana, I., Leendertse, K., & Handoko, W. (2013). Monitoring outcomes and impacts of capacity development in the water sector: a Cap-Net UNDP experience. *Water Policy*, *15*, 226–241. doi:10.2166/wp.2013.121

Holton III, E. F. (1996). The flawed four-level evaluation model. *Human Resource Development Quarterly*, *7*(1), 5–21.

Kirkpatrick, D. L. (1959). Techniques for evaluating training programs. *Journal of American Society of Training Directors*.

Kirkpatrick, D. L. (1976). Evaluation of training. In R. L. Craig (Ed.), *Training and development handbook: a guide to human resources development* (pp. 18–1 – 18–27). Mcgraw-hill.

Kirkpatrick, D. L. (1996). Great ideas revisited. *Training & Development*, 54–59.

Kraiger, K., Ford, J. K., & Salas, E. (1993). Application of cognitive, skill-based, and affective theories of learning outcomes to new methods of training evaluation. *Journal of Applied Psychology*, *78*(2), 311–328. Retrieved from http://psycnet.apa.org/journals/apl/78/2/311/

LaFond, A. K., Brown, L., & Macintyre, K. (2002). Mapping capacity in the health sector: a conceptual framework. *International Journal of Health Planning and Management*, *17*, 3–22. doi:10.1002/hpm.649

Lim, D. H., & Morris, M. L. (2006). Influence of trainee characteristics, instructional satisfaction, and organizational climate on perceived learning and training transfer. *Human Resource Development Quarterly*, *17*(1), 85–115. doi:10.1002/hrdq

McCauley, C. D., Ruderman, M. N., Ohlott, P. J., & Morrow, J. E. (1994). Assessing the developmental components of managerial jobs. *Journal of Applied Pyschology*, *9*(4), 544–560.

Mertens, D. M. (2005). *Research and evaluation in education and psychology*. Thousand Oaks: Sage.

Moore, M. L., & Dutton, P. (1978). Training needs analysis: review and critique. *Academy of Management Review*, *3*(3), 532–545.

Noe, R. A. (1986). Trainees’ attributes and attitudes: neglected influences on training effectiveness. *Academy of Management Review*, *11*(4), 736–749. Retrieved from http://amr.aom.org/content/11/4/736.short

Paradise, A. (2007). *ASTD State of the Industry Report*. *American Society of Training and Development*. Alexandria, VA.

Patton, M. Q. (2002). *Qualitative research & evaluation methods*. Thousand Oaks: Sage.

Rossett, A. (1987). *Training needs assessment* (2nd ed.). Educational Technology.

Tannenbaum, S. I., Cannon-Bowers, J. A., Salas, E., & Mathieu, J. E. (1993). *Factors that influence training effectiveness: a conceptual model and longitudinal analysis* (Vol. 642). Orlando, FL.

Yelon, S. L., & Ford, J. K. (1999). Pursuing a multidimensional view of transfer. *Performance Improvement Quarterly*, *12*(3), 58–78. doi:10.1111/j.1937-8327.1999.tb00138.x
