# Supplementary material for: Building capacity for water, sanitation, and hygiene programming: Training evaluation theory applied to CLTS management training in Kenya
Source: Soc Sci Med. 2016 Oct;166:66–76. doi: 10.1016/j.socscimed.2016.08.008 (PMC5034853; doi:10.1016/j.socscimed.2016.08.008)
Supplement: Supplementary file 3 [file mmc3.docx]

**Supplement 3: data collection tools**

Table of Contents

[Pre-training Questionnaire 1](#_Toc431645004)

[In-depth Interview Guide 1 2](#_Toc431645005)

[In-depth Interview Guide 2 7](#_Toc431645006)

# PRE-TRAINING QUESTIONNAIRE

Name of Participant___________________________________________________________________________

Current job title ______________________________________________________________________________

What department do you work in? ________________________________________________________________

What is the highest level of education you completed? ________________________________________________

In what year did you finish this education? (write year only) ___________________________________________

Have you attended any trainings or workshops for Community-Led Total Sanitation before? (circle one) Yes / No

Do you think the CLTS training workshop will help you improve your work? (circle one, and explain why)

Yes, because ______________________________________________________________________________

No, because _______________________________________________________________________________

What do you hope to learn during the next 5 days? (list) ____________________________________________________________________________________________ ____________________________________________________________________________________________________________________________________________________________________________________________________________________________________________________________________________________________________________________________________________________________________________________________________________________________________________________________________________

____________________________________________________________________________________________________________________________________________________________________________________________________________________________________________________________________________________________________________________________________________________________________________________________________________________________________________________________________________

# IN-DEPTH INTERVIEW GUIDE 1

Interview 1 is scheduled for two weeks after participants have been trained by Plan Kenya.

***Interview goals:***

1. Assess learning against curriculum learning objectives of the training:
   1. Knowledge of CLTS facilitation process, and triggering steps/tools
   2. Creating conditions for effective scale-up of CLTS, planning for scale-up
   3. Preparation and management of field facilitators
   4. Monitoring/reporting of CLTS activities and outcomes
   5. Partnership with government and NGO stakeholders
      1. Advocacy for funds
      2. Advocacy for technical assistance
2. Determine to what extent trainees’ expectations were met
3. Assess the value of the training to trainees (and how the training content and delivery could be improved).
4. See if trainees have identified how they could *apply* training in their job (or, how do trainees anticipate their work will change as a result of the training).
5. See how long trainees have been in their current jobs, and what factors have influenced their decision of what organization to work for.

**Interview begins here:**

***Introduction:***

Good morning/afternoon. My name is __________, and I work for Research Guide Africa. I am working on a research project with the (institution leading research). The purpose of this study is to improve training programs in Kenya and internationally. I am here to discuss with you and learn from your experiences. We are also interested in how useful the training led by Plan Kenya you attended is for you in the future. We will contact you again in six months for another discussion, even if you have changed jobs. This interview will last approximately one hour. This interview is private and confidential. I would like to record this interview to enable me to write a clear report from our discussion. The recording and any notes I take will only be used for the purpose of this research. The recording and notes will only be seen by Research Guide Africa and a researcher at the (institution leading research). When we write any reports, we will not include your name or identify you in any way. May we begin our discussion?

***Interview questions:***

“I would like to begin by asking you a few questions about your job…”

1. “Briefly, could you please explain your current role and responsibilities,
   1. “How long have you been in your current position?” (***Prompts****:* “How many years and months?”)
   2. “Can you give me an example of your current work?”
   3. “Who do you report to?”
   4. “Who do you supervise?”
   5. “Why did you take this job?”
   6. “What are a few things about your job that motivate you?”
   7. “What are a few things about your job that discourage you?”
2. “What is your previous work experience?”
   1. “What organization did you work for before this job/position?”
   2. “What county/district was that job/position in?”
   3. “What was your previous position?”
   4. “What were the reasons for the change of job?”
      1. “Did the March elections have any influence on your decision to change jobs?”
      2. “Did the change from districts to counties in March have any influence on your decision to change jobs?”

“I would like to ask you a few questions about the training program led by Plan Kenya that happened two weeks ago. Please think back to this training…”

1. “What part of the training program led by Plan Kenya had the most impact on you?”
   1. “Can you describe what you were taught during the training?” (***Note if the respondent can’t remember and needs to refer to written notes****)*
      1. “What was different for this training from other trainings you have attended?”
      2. “Can you describe the different steps in CLTS facilitation?”
   2. “Can you describe the activities and exercises of the training?” (***Prompt****:* “Do you remember any specific activities/sessions?”)
   3. “What did you like about the training?”
   4. “What did you dislike about the training?” (***Prompts****:* “Was there any part of the training that wasn’t useful? That made you uncomfortable?”)
2. “In what sort of community do you think CLTS would work best?” (***Prompt****:* “Are there any characteristics of a community that would make CLTS more likely to succeed?”)
   1. “Do you think CLTS can be successful in your county *(or other jurisdiction*)?”

***If no****:*

- 1. “Why not?”
  2. “What do you think would need to change in order for CLTS to be successful in your county (*or other jurisdiction*)?”
  3. “Is there anything about your country (*or other jurisdiction)* that would make CLTS successful?”

***If yes****:*

- 1. “What characteristics of your county would help make CLTS successful?”
  2. “What challenges are there for CLTS in your county?”

“Now I would like to ask you a few questions about your current job in detail…”

1. “Do you manage/supervise staff?” *(****Prompt****:* “Do you oversee or manage anyone at your organization?”)

***If yes:***

- 1. “Can you tell me what this involves?”
  2. “Who do you manage/supervise?”
  3. “What tasks are they responsible for?” (***Prompt****:* “Does anyone you supervise work with the communities?”)
  4. “How do you delegate tasks to them?” (***Prompts***: “How do you do workplanning, scheduling, and organizing tasks for people you supervise?”)
  5. “Since the training by Plan Kenya, will you manage/supervise staff or delegate tasks differently?”
     1. “Why or why not?”
     2. “How will you do this differently?”
  6. “Do you plan on doing anything innovative or new in managing/supervising staff”

1. “Have you done any monitoring of project progress or outcomes?” (***Prompt****:* “What projects?”)

***If no****:*

- 1. “In your previous job?”

***If yes****:*

- 1. “What did this monitoring focus on?” (***Prompt****:* “What information do you collect?”)
  2. “How did you do this monitoring?” (***Prompts***: “Who collects information, how often, at what stage?”)
  3. “What is the purpose of this monitoring?” (***Prompt****:* “What is the monitoring data used for?”)
  4. “Since the training by Plan Kenya, will do monitoring differently in the future?”
     1. “Why or why not?”
     2. “How will you do this differently?”
  5. “Do you plan on doing anything innovative or new in monitoring?”

1. “Have you done any reporting on projects’ progress or outcomes?”

***If no****:*

- 1. “Do you plan on doing any reporting in the future?”
     1. “Why has this changed?”

***If yes****:*

- 1. “Who were you reporting to?”
  2. “What did this reporting focus on?” (***Prompt****:* “What information was in this report?”)
  3. “How do you do your reporting?” (***Prompt****:* “Is this how you’ve always done it? What format do you use for reporting?” “Is this how you’ve always done it?” ***If no*** “In the past how have you done this reporting?”)
     1. Is this how you’ve always done it in the past?
  4. “Since the training by Plan Kenya, will you do reporting differently in the future?”
     1. “Why or why not?”
     2. “How will you do this differently?”
  5. “Do you plan on doing anything innovative or new in reporting?”

1. “Have you worked on any projects involving partnerships?” (***Prompts****:* “Such as NGOs, private businesses, or other government departments?”)

***If no****:*

- 1. “Do you plan on working with partners in the future?”
     1. “Who do you plan to have partnerships with?”
     2. “What will be the role of the partner?”

***If yes:***

- 1. “Who were these partnerships with?”
     1. “What did these partnerships involve?”
     2. “What was the role of the partner?”
  2. “Since the training from Plan Kenya, do you plan on working with partners differently in the future?”
     1. “Why or why not?”
     2. “How will you do this differently?”
  3. “Do you plan on doing anything innovative or new involving partnerships?”

1. “Can you think of a time when you had to request for funds for projects in your county (*or other jurisdiction)*?”

***If no****:*

- 1. “Do you plan on requesting funds for your projects in the coming year?”
     1. “From whom do you plan on requesting these funds?”
     2. “How do you plan on doing this?”

***If yes****:*

- 1. “Have you ever had to ask for an increase in funding for a project?”
  2. Have you ever had to ask for a change in funding for a project?”
  3. “What project were you requesting funds for?”
  4. “From whom did you request these funds?” (***Prompts****:* “From your supervisor? Partners? Someone else?”)
  5. “Can you tell me how you went about doing this?”
  6. “Since the training by Plan Kenya, will you make requests for funds differently in the future?
  7. “Why or why not?”
  8. “How will you do this differently?”
  9. “Do you plan on doing anything innovative or new in requesting funds?”

1. “Can you think of a time when you had to request for more technical assistance for projects in your county (*or other jurisdiction)*?”

***If no****:*

- 1. “Do you plan on requesting technical assistance for your projects in the coming year?”
     1. “From whom do you plan on requesting these resources or support?”
     2. “Why has this changed?”

***If yes****:*

- 1. “What project were you requesting technical assistance for?”
  2. “From whom did you request this technical assistance?” (***Prompts****:* “From your supervisor? Partners? Someone else?”)
  3. “Can you tell me how you went about doing this?” (***Prompts****:* “How did you decide who to ask?”)
  4. “Since the training by Plan Kenya, will request technical assistance differently in the future?” (***Prompts****:* “Would you go to anyone different?”)

1. “Why or why not?”
2. “How will you do this differently?”
   1. “Do you plan on doing anything innovative or new in requesting technical assistance?”
3. “From the training led by Plan Kenya two weeks ago, are there any lessons you would like to pass on to your colleagues in your department or ministry?” (***Prompt:*** “Any specific lessons?”)

***If no****:*

- 1. “Why not?”

***If yes****:*

- 1. “What CLTS principles would you like to pass on to colleagues?”
  2. “What management skills would you like to pass on to colleagues?”

(***If they give short responses, ask for more details****)*

# IN-DEPTH INTERVIEW GUIDE 2

**For trainees who *have NOT changed* position or job since the training**

Interview 2 is scheduled directly after mentoring, approximately 7 months after the training by Plan Kenya.

***Interview goals:***

1. Assess the perceived value of the mentoring
2. Assess how their activities have changed during mentoring and whether or not any of these changes will carry forward after mentoring is finished.
   - Supervision
   - Partnerships
   - Resource mobilization
   - Monitoring
3. Determine if and how trainees have attempted to pass on their learning to others
   - CLTS knowledge
   - Management skills

***Instructions to the interviewer:***

- If an interviewee gives a vague description, ask them if they can provide a specific example. This is especially important for:
  - “Why or why not?” questions
  - “How…?” questions
- Make sure to ask *every* question unless a question is in parenthesis
- Ask all primary questions the way they are written (questions numbered 1, 2, etc.)
- Add notes on body language (e.g. head shaking, nodding, excited hand gestures, etc.) to the interview transcripts.
- Continue to record interruptions in the interview and laughter in the interview transcripts.
- **Skip to question 3 at 15 minutes into the interview if you have not already gotten to question 3**
- **Skip to question 7 at 35 minutes into the interview if you have not already gotten to question 7**

**Interview begins here:**

***Introduction:***

“Good morning/afternoon. My name is __________, and I work for Research Guide Africa. I am working on a research project with the (institution leading research). You may remember that we interviewed you last year, when you consented to be in this study and gave us permission to contact you in the future. Your last interview was very valuable and we would now like to interview you again. This interview will last approximately one hour. We will contact you one last time in twelve months for another discussion, even if you have changed jobs. I would like to record this interview so that I can write a clear report from our discussion, but you may ask that the recorder be switched off at any time. This study is completely voluntary, and you may skip any questions or stop at any time. This interview is private and confidential, when we write any reports, your name and identity will not be included in any way. May we begin our discussion?”

***Interview questions:***

1. “I would like to begin by asking you a few questions about your job…”
   1. “What are a few things about your job that motivate you?”
      1. “Has this changed in the past six months?” (***Prompt***: “How has this changed?”)
   2. “What are a few things about your job that discourage you?”
      1. “Has this changed in the past six months?” (***Prompt***: “How has this changed?”)

“I know you have been mentored over the past 7 months. I would like to ask you a few questions about the mentoring program led by Plan Kenya that you have participated in.”

1. “Can you briefly describe the mentoring program you went through with Plan Kenya?” (**Allow 2-3 minutes**)
   1. “What were your expectations for the mentoring program?”
   2. “What part of the Plan Kenya mentoring program did you find the most interesting?”
      1. “Can you give a specific example?”
      2. “Why did this stand out to you?”
   3. “How could the mentorship experience have been improved?”
   4. “Was there any part or any specific activities of the mentoring that wasn’t useful?” (***Prompt***: “Was there any part that made you uncomfortable?”)

(**At 15 minutes, skip to question 3)**

“Now I would like to ask you a few questions about your current job in detail…”

1. “Tell me about your approach to managing or supervising staff.” (***Prompt***: “Tell me about your management style.”) (**Allow 2-3 minutes**)
   1. “Over the last 7 months, since the training by Plan Kenya, have you managed staff, supervised staff, or delegated tasks differently?”
      1. “Why or why not?”
      2. “How have you done this differently?” (***Prompt:*** “Can you give a specific example?”)
   2. “Will you continue to do supervision differently in the future, now that the Plan Kenya mentoring program has ended?”
      1. “Why or why not?”
2. “Tell me more about the partnerships you have been involved with, including interdepartmental and NGO partnerships.”
   1. “How would you describe your relationship with these partners? (**Allow 2-3 minutes for 4 and 4a**)
   2. “Are any of these partnerships new in the last 7 months?”
   3. Over the last 7 months, since the training by Plan Kenya, have you worked with partners differently?”
      1. “Why or why not?”
      2. “How have you done this differently?” (***Prompt:*** “Can you give a specific example?”)
   4. “Will you continue to work with partners differently in the future, now that the Plan Kenya mentoring program has ended?”
      1. “Why or why not?”
   5. “Will you start any new partnerships with other departments or NGOs in the future?”
      1. “Why or why not?”
      2. “With which departments/NGOs?”
3. “Tell me about your work on resource mobilization in your sub-county/county?” (***Prompt***: “Such as requesting funding, or writing a funding proposal?”) (**Allow 2-3 minutes**)
   1. “Over the last 7 months, since the training by Plan Kenya, have you worked on resource mobilization differently?”
      1. “Why or why not?”
      2. “How have you done this differently?” (***Prompt:*** “Can you give a specific example?”)
   2. “Will you continue to work on resource mobilization differently in the future, now that the Plan Kenya mentoring program has ended?”
      1. “Why or why not?”
   3. “Will you start any new projects in the next year?”

***If yes****:*

- - 1. “Do you have a specific project in mind?”
    2. “How will you mobilize resources for this project?”

1. “Tell me about your monitoring of project progress or outcomes.” (***Prompts****:* “What was the purpose of this monitoring?” “Why do you monitor progress or outcomes?”) (**Allow 2-3 minutes**)
   1. “Over the last 7 months, since the training by Plan Kenya, have you done monitoring differently?”
      1. “Why or why not?”
      2. “How have you done this differently?” (***Prompt:*** “Can you give a specific example?”)
   2. “Will you continue to do monitoring differently in the future, now that the Plan Kenya mentoring program has ended?”
      1. “Why or why not?”

(**At 35 minutes, skip to question 7)**

1. “Currently, how do you think your sub-county/county is doing in terms of CLTS?”
   1. “Do you have any plans to expand the scale of CLTS activities in your sub-county/county?”

***If no****:*

- 1. “Why not?”
  2. “What challenges does your sub-county/county face in scaling up CLTS?”

***If yes****:*

- 1. “Do you think your sub-county/county can scale up CLTS without help from Plan Kenya?”

***If no****:*

- - 1. “Why not?”
    2. “What challenges does your sub-county/county face in scaling up CLTS?”

***If yes****:*

- - 1. “What is it about your sub-county/county that will make CLTS scale-up successful?”
    2. “What challenges does your sub-county/county face in scaling up CLTS?”
    3. “How has the training and mentoring from Plan Kenya helped you to scale up CLTS?”
    4. “Can you give a specific example?”

1. “Think back to the training and mentoring by Plan Kenya, what would you say are the most important lessons you have learned from them?”
   1. “Have you been able to pass any of these lessons on to your colleagues in your department or ministry?”

***If no****:*

- 1. “Why not?”

***If yes****:*

- 1. “What CLTS principles or knowledge have you tried to pass on to colleagues?”
     1. “How did you pass this information on?”
  2. “What management skills have you tried to pass on to colleagues?”
     1. “How did you pass these skills on?”

(***If they give short responses, ask for more details****)*

- 1. “Why did you decide to share these lessons or skills with your colleagues?”
  2. “How do you think these lessons or skills will help your colleagues improve their work?”

1. “Has the training and mentoring program by Plan Kenya influenced your work in any other way?”
