# Supplementary material for: Building capacity for water, sanitation, and hygiene programming: Training evaluation theory applied to CLTS management training in Kenya
Source: Soc Sci Med. 2016 Oct;166:66–76. doi: 10.1016/j.socscimed.2016.08.008 (PMC5034853; doi:10.1016/j.socscimed.2016.08.008)
Supplement: Supplementary file 4 [file mmc4.docx]

| Box | Sub-box | Quote |
| --- | --- | --- |
| Learning outcomes | CLTS process and steps recall | “the Pre-triggering, there is first sensitization and the community is brought on board and then various people are on board and a meeting is done and then there is mobilization. In that meeting the real triggering is done and people are asked to look for a stool in the village they do mapping and identify the houses and they identify that they were not using latrines before and be able to identify their problem and they want to try to educate and then go out there and educating and exposing the people to the stool and practically show that it’s not a good thing and then as well and bring water and bread and put stool in water and shake it and ask if any of them could drink. All of them will refuse and this is to make them disgusted and ashamed that they should not be associated and their minds are triggered. You see when see a different perspective for them when they now see that when you they leave a stool somewhere they don’t think about it and so when you demonstrate that when the rains come the shit will go into the river, they will be able to see that what they are doing is a really bad thing” |
| Learning outcomes | CLTS success and challenge factors | "We never thought that the [county name] community would embrace CLTS as it has done. Because of the culture but we are seeing the culture is really not a big issue now" |
| Learning outcomes | CLTS success and challenge factors | "when you use the Adult teachers who are locals who live in those villages will help in the triggering process very well like [place]." |
| Learning outcomes | CLTS success and challenge factors | "The other challenge we have….although the issue is being addressed now. Squatters. You know it is difficult for a squatter to put up a structure and then put up maybe a toilet. There it will be a challenge. Because the owner may say, this is not your land. You cannot do this." |
| Learning outcomes | CLTS success and challenge factors | "The pampus [disposable diapers] issue is a big challenge! And it gives me sleepless nights as I have been saying it everywhere to myself be we really an issue because when you see pampus you are seeing shit! Nothing else...We have worked on a bill and that bill is yet to be passed by the county assembly...It is a bill on waste management where we want to further ban the issues of careless disposal of pampers and the littering issues and total waste management and if this is approved we then would go to villages and urban areas trying to explain what this bill is all about and once in force I think some of these problems will be solved." |
| Learning outcomes | CLTS success and challenge factors | "The only part is the mentoring program is when we come up with the budgeting during triggering the facilitator or the team should really work with the local scenarios which are there like the effect of not having a latrine and the budget for one who falls sick because of not having a latrine .The facilitators should really zero down on things that are happening in that village and at times issues may vary from one village to another .That one should be improved as per on that particular site They should not ask standard questions as they do?...Maybe they should ask ‘what is the cost of a mosquito net or a drug’? Or for example the costs of incurred when you are taking a dead body to the mortuary and use the local situation understanding culture and religion of the people and trigger or train whilst taking care not to upset status quo .For example you have gone to a village and it is an Islamic village the moment that a person dies he is buried 2 or 3 hours later and so when the facilitator starts probing ‘so how long do you take in preparation for body to be buried doesn’t apply to them because zero costs are inured ...Or you can also be in an area where they are neither Christians nor Muslims but after burial they stay for 7 days and feast and do shit and at the same time there is no latrine .So from there during this time or for the duration that these guests are here they have a negative impact on the open fields where they defecate .People then become sick after one or two weeks and may even be hospitalized. So my point here is giving the people local examples that are happening in their area so that they can identify with them and come up with local solutions. That is the essence of CLTS – community-led total sanitation." |
| Learning outcomes | CLTS success and challenge factors | "Because we realize…I understand there are some villages that had already been declared ODF, but then at times they also revert back. So that could be the biggest challenge. You know actually changing their mind set and changing them from whatever they…you know there are some cultural believes that will be hindering that, to achievement of CLTS. So that is what could be a major challenge." |
| Learning outcomes | Potential partnership skills | “I would say I have worked differently because out of the training I was trained on the 4 Ps of the partners. That is the Policies, the Priorities, the Procedures, and the Psychology of each and every partner. So out of that I can be able to get to the partner very well if I have already known all their requirements.” |
| Training design | Bangladesh video | "yes there in Bangladesh they were using the Bamboo to prevent the soil from collapsing, here we can use the Guava trees or stems to control the soil from collapsing. We also say that when you dig a round pit its more stable compared to the rectangular pit." |
| Training design | Bangladesh video | "Especially the Bangladesh one is the one that really tried to show us who were new to the triggering process. It was Professor Kamal going to identify the faces and where the community members would go to defecate and understand that by doing that it created that whole scenario of diseases. This video is the one that tried to show us that CLTs requires joint collaboration between the facilitator and the community. We don't have to think of materials from outside they used were local materials like they were weaving those bamboos and also for the supporting structures they got local wood planks. For me as a civil engineer my area I can use local materials like I can weave the local trees/bushes to make bamboos to make the for water tanks. I found that we don't need the weld mesh because it’s expensive and we can weave form our local trees and use them comfortably and prevent the soil from collapsing. In the video there was the use of local resources, local skills and the human labor is available as well as the interaction between the facilitator and the community." |
| Training design | Bangladesh video | "At least from the video I learnt that you can actually sensitize the community by you know involving them you see you involve them in what they do on a day to day basis and educating them at the same time. So that they can realize that actually there is a problem within their community and also you somehow give them the opportunity to come up with solutions on the same problems they have actually identified." |
| Training design | Field experience | "I liked many things and For me that field trip was the best it gave me an opportunity, to see like you see like when the Public health officer demonstrated those things physically and when he took a stick placing it into faces and inserted stick into faces and put in water and people are reacting that how are we eating faces and they got triggered that kind of reaction that was very striking and I liked it very much" |
| Training design | Field experience | "The positive thing that I learnt there was restrain because as the triggering process was going on there are things that I saw were going haywire but you just remain calm and you can’t intervene when the session is going on because it will embarrass the facilitator so a manger /supervisor needs to just watch and see and not intervene you reserve comments to be discussed later in the review. By not restraining yourself and coming out to correct a facilitator you intimidate him and he will be looking at him/herself and will always be uneasy when you are around waiting for other inputs from you as the officer all the time...Those who attended this training for the first time were they saw the mapping exercise and even the transect walk. They commented and said it would be good to blend the women, children and men or adults so that we be shown the areas where they openly defecate. we even discovered that what they said was true because the groups that went with the adults took longer as they kept meandering and not taking us where the faces were but the children who were with another group too them straight to the areas . After that one of the participants said that if we had gone with the children we wouldn’t have taken that long the adults were not telling us the truth." |
| Training design | Field experience | "the first challenge was not so many members attended the triggering exercise we had discussed this even in our sessions that proper sensitization is required before the actual triggering day. One of the things I noted is that there is need to work closely with the Provincial Administration, the Opinion leaders, and also walking there earlier and walking around physically in the area to see if that problem exists and of course familiarizing yourself with the area. We went there and the reason why there were fewer people is that they changed the village that we went to work on at the last minute. This probably was because the area they had earlier chosen had been triggered this means the person going to trigger must personally walk to the field and at least see and know the village where we are going to trigger is it the appropriate place to trigger or not. We noted that." |
| Training design | Field experience | "What should be improved is the field trip we should not have only one field trip because the one we went for the community didn’t want to co-operate with the facilitators, they didn’t turn out in large numbers and we found that they knew what was going to happen and even were saying that ‘you will be shown faces’! You know that the community was aware of the triggering process and we found out that this one was not coming out clearly and the issue of shame and disgust was not really coming out so if we had another community or village to visit then we would have heard a comparison or maybe get the right result. At times also the change of facilitators, maybe the facilitators’ approach would affect the people’s reaction and therefore there is a need to change the facilitators. We can even have the males alone and women alone and the children alone and see if we can learn better ways of improving the triggering process. Maybe if its gender specific the reactions maybe different and people will react differently. Or even after that we may realize that it’s good after all to just mix the two of them together " |
| Training design | Cross sectoral | "We have programs in the Ministry of Agriculture, we have been having like NALEP, but the CLTS program approach was really a bit unique in a way that it dealt with the community member himself. So improvement maybe is to bring on board more of the actors. Even though they had involved other NGOs but mostly it was departmental heads. And mostly as a County, you find that all the departments depend on the government funds, or the County funds. But bringing on board the other NGOs like the World Vision, let us say Action Aid, AMREF, if they have the aspect of community health, they would assist because we need funding. We have to fund raise for funding. So bringing the other actors on board will also give it a lot of strength and more success can be seen." |
| Training design | Participatory | “even the sitting arrangement was bringing everybody at equal level whereby it was a semi-circle as I said from the start, and you find like we were sharing at the same level, you know there were people there like the DC’s you know we rarely share with them at the same level though we are departmental heads but you know they enjoy certain privileges that we don’t enjoy. So in that scenario we were like in the same wave length and we could interact freely and share freely.” |
| Attitude and motivation | Reasons for taking current position | "I used to admire the public health officers as a young girl. I used to have a neighbor at home who was a public health technician. She used to visit our households and she used to be called the cleanliness woman, so I used to admire her when she was walking around the village and everybody really respected her. So it’s not really a job I really knew what it was all about apart from the fact I knew, I would see her visiting, going around on a bicycle and everybody would say the cleanliness woman is coming, and it is a long time ago." |
| Attitude and motivation | Motivators/ discouragers | "Or you find that one person jumps from this training to another training or another training and leave others, sometimes you get demotivated." |
| Attitude and motivation | Motivators/ discouragers | "When I go to the field. Not when I am in the office. Maybe I go to do a project or to train. That motivates me. When I have to stay in the office, I do not like it….Maybe, even the way the government operates. You are not supervised directly. There is no one who keeps an eye on you all the time. You are somehow independent." |
| Attitude and motivation | Motivators/ discouragers | "Of course I am more energized because of the fact that more than the basic training I received as a health officer I have had an opportunity to receive additional trainings and that really shapes me up (passion) and it has focused me to do my work very well. In the last six months we have seen that we are trying to move faster than we used to do and that is encouraging because you see when you get results you really feel that you are now on track (excited) and you feel more energized to work harder !" |
| Attitude and motivation | Motivators/ discouragers | "It is an interesting job. It is scientific, it is innovative, and also there is a lot of introduction to the community people. You learn a lot and exchange many ideas. We don’t basically depend on what we learned in college, but we always try to come up with new discoveries, innovations, planning for the community and it something which you have to also continue learning, because different recommendations come up from time to time. Like sometime back we were using DSP as our fertilizer in our soils, but recently when we took some soil sampling, we found that we needed a lot of NPK. A lot of K and Phosphorus. It is something we have to research from time to time." |
| Attitude and motivation | Motivators/ discouragers | "As per the work I do particularly the way the Policies come from the top rarely give chances to the community to participate of being Total decision. They have resources but the decisions are made up there by the Policy makers. For me this is discouraging" |
| Attitude and motivation | Motivators/ discouragers | "No! I can’t reverse Rose!! I can’t reverse!! I told you that I have passion for my job earlier and so reversing would mean that I have lost the passion and I don’t want to lose the passion because this mentorship project is over. You know I have! That is why I say that I have a big burden in the community because there are so many programs taking place in the community and they are projects they have a start date and an end date. And always worries me if a project ends what will then drive the community and there now you will need the service of the health promotion officer, everybody will leave. Every other department will say ‘that Plan thing came to an end and we are waiting for it if it comes back! But now as a health promotion officer it is my burden to see that this continues , enablement of peoples health issues continues so what has been started it has to move on ! It is my pain to find out how it moves on in some way small or big with or without funding it has to somehow move on. It may be difficult because we unfunded but it is the prerogative of my office to ensure that people’s health is improved...Remember that health promotion deals with environmental enablement and so if I pack up and say that ‘Plan mentorship went! EGPAF went! UNICEF went! Then I will be killing the community!!(Passionately stated) |
| Attitude and motivation | Motivators/ discouragers | "The few things that motivate me in my job, you see like I had said we are community focused and therefore something which motivate me, like I once worked in [Place] before I came here so even a few weeks ago some people, staff there and clients were ringing me “[Trainee's name] so you were getting as through on a wonderful way, you are like an eye opener to us you have made us an inch different from the other staff. I feel comfort and satisfied in myself and think if I have touched souls and create this kind of change, not only to the people that am supervising but now to those people who are supposed to be the beneficiaries the clients in the community down there, therefore in one way or the other I will be satisfied and feel that am at least doing something." |
| Attitude and motivation | Motivators/ discouragers | "For me what motivates me about this job is when someone succeeds and they tell you when we are in a discussion session or a sensitization session and they tell you that from their discussion you get that their attitude has changed for better response towards something to is more positive because most of the time our work in mainly on sensitization and training and am happy when there is a positive change in someone’s life." |
| Attitude and motivation | Motivators/ discouragers | "Also I can say the personnel that are the interaction was more frequent as compared with other departments and it’s very easy and we are within we can plan easily and forge very well. Another thing that motivated me was In the water sector 2002 there was the policy that in every district there must be a water environmental sanitation co-ordination committee called WESCOD and I have been trying in all districts that all have WESCOD committees. When I was appointed to [county name] I was really motivated because now my performance will be better because I will concentrate so much on one county compared to the vastness of the area and monitoring it in as far as water and sanitation is concerned." |
| Attitude and motivation | Motivators/ discouragers | "In fact like the Ministry of Agriculture who even went as far as look for resources to implement CLTS by themselves and as managers we did trigger as managers the villages though it was attended by 8 villages like here you can talk of a sub location where the mangers themselves are doing a follow up themselves...The department of agriculture, the water department, the children’s department, the youth department and we are there as health department and that is motivating me because if the other departments are coming to the forefront that makes me always want keep up with the pace." |
| Attitude and motivation | Motivators/ discouragers | "The coordinating role also motivates me because you are able to get to know what others are doing unlike if you are alone and you don’t really participate in these…Because for me I can almost tell you what is happening in other departments, but if you went to another department they might not be in a position what is happening in health, what is happening in OP, what is happening in the County Government. So you see that coordinating role makes you like a center person, this is like a referral point. Somebody can come here and can be directed on what is there, what is happening. Even the NGOs when they come, the entry point is normally this office. They will come and you know, tell us they exist, and then I will guide them where they are supposed to go next. Like in those committees that we have, like if you wanted to work here, of course we will invite you to one of the committees where stakeholders sit, of course they will get to hear what you do. So again this is like an entry point when you come to the district, because everything is development." |
| Attitude and motivation | Motivators/ discouragers | “Another thing that motivates me in this job is that we have varied experiences and thinking dimensions in the community, so across [Trainee's county] I can say I openly know how [sub-county] behaves, I can also say I know how [sub-county] behave and comparatively I can use this experience to interchange to this, it has made me have diverse thinking when it comes to meeting people at community level and it has helped me know how to intervene.” |
| Attitude and motivation | Motivators/ discouragers | “then Lake Basin came up, that is 1992, and when it came up I found the public health officer and community health extension officer, the social services people, were orators and here I was somebody who was still government-oriented giving orders, do this do that, so it was a big challenge to me but when I saw the way they were working. I admired the way they were working so we had a mutual, I opened up my heart to them and told them “the way you are moving about with the community, I admire and I want to emulate that. As for now, I don’t have that language so just help me to help you so that we achieve”. So they understood my problem and predicament and agreed to help me. So in that situation we could have a compromise whereby where I was supposed to talk, they talk on my behalf but where there’s a technical issue I give the input. So it is them who nurtured me such that they removed the ordering part such that I can now face the community and discuss with them. So out of their team-work or out of their willingness, they molded me.” |
| Ability |  | No quotes for this section |
| Individual Performance Outcomes | Critical thinking | "When you look at the topics like community entry those are not technical things like how to identify and enter into a community and community accepts you. You know community entry is very important because you may say they are receptive and they tell you to come but when you go they just give you audience and once you go back they don’t do anything. For me what we really need more focus on is The Community entry methods and the challenges which we have so far. Like the community we have here what are the best methods for us to use to enter into these communities so that they can embrace our ideas, which target group to use do we use young people and the children to be the Change agents in the Community." |
| Individual Performance Outcomes | Monitoring | “under CLTs they have to form a committee so when I go down there I even meet them with those committees in the community and so I not only go to my centers or classes I also visit the committee members to get updates on sanitation.” |
| Individual Performance Outcomes | Monitoring | "Not really much difference.it is just the same. We have guidelines that we have always followed when doing monitoring. We have tools that we always use for monitoring" |
| Individual Performance Outcomes | Monitoring | "Monitoring we will continue in the same way it won’t change because Monitoring is standard and it’s a continuous activity because we need to know how far have you gone? How far and why and have reached your targets and that is what monitoring is all about." |
| Individual Performance Outcomes | Monitoring | "I’ll do change because even even...when CLTS, when CLTS works out well, then you see the results of reduction of diseases like the fecal oral diseases. So, I think it will help because we`ll compare the notes and compare the…which village, this village, which village is, diseases are now coming down, which village is this? Was it really a, have they put all the indicators in place, is it the reason to why these diseases have come down? Or if these cases are nini what is happening? So you see, you will link the two, the sanitation part of it and the trends?" |
| Individual Performance Outcomes | Partnership | “mentoring bonded us so much that nowadays when you are calling colleagues for an activity these are people that you are already working with and you are comfortable with them...recently a new NGO was coming in to collect baseline data…I simply cross over to [name] here at water office and he immediately knows what kind of information I need” |
| Individual Performance Outcomes | Partnership | "The way we are looking and approaching those people is now different. Because we go and sit down and discuss, and tell them why we are inviting them. So you explain. You don’t just send a letter. You discuss and they also tell you if they want to be on board and by the time they are coming on board, it is a win, win situation. So that is the change that we are anchoring or introducing to them or we are sharing with them." |
| Individual Performance Outcomes | Partnership | "Our relationship is more strengthened in terms of sponsoring workshops and they are now more involved and they are like now we have so many training needs and together with them we identify the gaps and then together we see how best they can support us. This never used to happen before it was just us being invited to a workshop without being involved in planning as per identified gaps or needs." |
| Individual Performance Outcomes | Partnership | "if an NGO has a program and it has scheduled activities that you have to be at this level at this particular time and then actually we might have a difficult time because we planned for an activity together but they have again make dates for the same and then communicate back however we have heard a rough time and what I said is that we have to plan specific dates for each and every other activity together not just the NGO coming and saying that they have already planned and so they say they want you to go there! So with NGOs we have had a difficult time and the other thing is that the clashing periods like whereby we planned for an activity like next week with Plan, Amref or what? And then it reaches a particular time like now we have a national activity like the polio activity that is coming and so you will not prioritize the activity that you had agreed on with the NGO because this is a regional activity and we must plan and fit with the national activities." |
| Individual Performance Outcomes | Partnership | "Of course yes. In the first place why did I attend that mentorship program? It’s because I wanted to get new ideas that I can also use elsewhere. So an example is this program that we are now doing, what we are going to do today, this planting of trees. You know if it were not for this mentorship by Plan, I wouldn’t know how to approach these KFS guys, and tell them, ‘you know, we have to do this, we have to conserve our environment and you have been noted as stakeholders.’ So maybe I would have done it but it wouldn’t have come out so well, or maybe I would have done it but it would have failed, but I thank God that it went through" |
| Individual Performance Outcomes | Resource mobilization | “You see like last year when we approached the county government they had not understood what we were doing so this year we started off by calling them into a sensitization meeting and we explained to them what we are doing and what we have achieved, the bottlenecks and how they can come in and you know also in resource mobilization you need to focus somebody in what part is that player taking? Is he doing the entire thing? Or is he just doing a section of it? So we were are able to bring in the county government and explain to them the role we wish them to play and I think they appreciated and I think now they have promised us viable pledges, you know really tangible pledges that maybe starting in July when we start next financial year and so we should be able to now to move better. We can now say that we are now having that assurance that they will support sanitation in [Trainee's sub-county and county] as a whole. I am sure before the training we had not focused ourselves properly but right now we are better focused to handle it.” |
| Individual Performance Outcomes | Resource mobilization | "Like, I think after attending that training, it enabled me like 2 or so weeks ago, having attended the training in [Place], it enabled me us to… I wrote some concept note to Action Aid and they accepted and funded training for the youth in [Trainee's sub-county]." |
| Individual Performance Outcomes | Sanitation activities | "I used to have a (laughs looking at a hanging that was not on his wall) I think someone removed it. I used to have something about (chieth!!)- Faces in dholuo and I would be asking people when they come to register groups and I would ask them ‘Do you have a toilet? Or are you eating chieth!!- Faces like that and I think the word became too much and they must have removed it (laughs). But aah! It has enabled me mobilize people whenever they come to register I bring up that issue of constructing sanitation units, I mean toilets as a project which they should be having as part of their groups. So that one has actually helped me in sensitizing groups as they register previously I would not have thought that toilets were that important but personally after the mentorship program my mind has changed. I can say that toilet is good that is ‘a home without a toilet is inhabitable!" |
| Individual Performance Outcomes | Sanitation activities | "Maybe not in the way it has been modeled but as an irrigation I have a contribution to make because since I attended those trainings I have influenced two project designs to include toilets as part of the design of the project so that when the donor comes to fund he also funds the construction of public toilet units as well...In [place] there is a project called [project name] previously we used to make the designs for the infrastructure and nothing else but now we have included in the BQ’s the toilets to be constructed as part of the BQ’s....In [place] a project called ‘degu degu’ we had training there and as I was training the farmers and I brought out this issue of CLTS and they actually agreed that they were now going to toilet pits. I mean pit latrines!!" |
| Individual Performance Outcomes | Supervision | "A combined approach system is a system whereby like you know we have the CLTS team and we are partners and then we have the community .This combined approach system is whereby at least every quarter of every month we develop a few people under the mentorship. One is a community member, one is a team member and one is the implementing partner member. I remember one time when we engaged the governor and we do evidence based practice under combined strategy where the problems and solutions are stated by the community .The evidences at the management office are stated by the manager and the implementer states his side and this is the combined approach system where nobody speaks for the other but you are all at same level and undertaking the same project but are free to give objective findings or reports." |
| Individual Performance Outcomes | Supervision | “I can say conflict management nowadays we have a small management system within or in-house and I really got some good skills from the mentoring program…Like coming to a dialogue on any issue I can give an example of bringing two colleagues who are conflict come to my office and I order for tea so that they take together and you actually can see that they don't want to take this tea and so eventually I serve them the tea and the tension is removed and bonding starts as I talk to them together over a cup of tea. Actually the best way for conflict management is dialogue.” |
| Attitude and motivation | Motivated by personal interaction | "I like my job because I work with many people. I interact a lot with people. I just enjoy working with people, talking with people, interacting with people, and also trying to know how other people live. So I really enjoy working with communities, it takes me down to the people on the ground and also our office by extension. We call it a social office, it is very friendly. It is open to all; everybody is comfortable and we also, one of our target group is vulnerable people. People with disabilities, elderly, the young, the women, and the marginalized so to speak of some of those people you; they really feel comfortable working with them. We give them that human face and by nature of our training, those are some of the issues we address and we do a lot of counseling. We do a lot of counseling and training. We have done a lot of talking with whoever wants to talk to us." |
| Attitude and motivation | Motivated by personal interaction | "One thing that is a motivating factor is a very receptive community here in [Trainee's county]. At first when I was posted I did not want to come here but when I was appointed here in [Trainee's county]. By then my colleagues were telling me that those people are difficult, they don't want to change from their culture and so many issues, but when I came here I stayed with the community, I took my time to learn from them and they also learnt from me. So I learnt that those who were saying the that the community was not receptive maybe it is the officers were there that had the problem but not the community. We could be the problem but honestly from this community I went to each and every other location and I sat with the leaders and the community members and I had a one on one discussion and from that I learnt that if the community entry were not properly followed then you experience some difficulties but if you use good community entry points then the community will accept you and take whatever you tell them and you move along with them." |
| Attitude and motivation | Motivated by personal interaction | "I found it very enjoyable to interact with community members, the older persons they teach me a lot." |
| Attitude and motivation | Motivated by personal interaction | "One other thing its continuously building up the capacity and it’s the approach of knowing Behavioral characteristics of people and it has the Socio-cultural characteristics where they communities directly meet you. You can exchange with them, you can gain more knowledge so that gives more interest in sharing with people." |
| Ability | Healthy populations | "Actually I added it to my priorities as a health education officer because it addresses the core function of health promotion. Health promotion is different in this way from other health care. One sits by the riverside and sees the others drowning he will help by either trying to get them out and he gets overwhelmed he rescues one and then he rests a little and then he sees another one taking a bath and is drowning and he rescues and then another one coming he again rescues and that is what health care is addressing. But health promotion you will have to go up the river to find out why people are drowning and then as a health promotion officer you have to find how to put up a bridge there so that people can cross and not slip into the river and drown. The health care which gives the medical part of it waits for them downstream to resuscitate them and let them go back home. They will let them go back home and they will again try and cross the river and drown and again come back here. So health promotion is like going up there and address why people are drowning and so CLTS is one way of addressing why people are drowning. Health education is one way of helping to see why people are drowning. You give them knowledge and you tell them here is slippery you need a bridge here. You work with them together to build up that bridge. Once the bridge is built level 6 which is the rescuer will be relaxing because there will be no people drowning because people will be crossing. So CLTS adds to that and that is why I give it a meaning in my work and my priorities as a health promotion officer. I involve everybody in the level of health and it is through persuasion because some say ‘Oh! Am burdened by this commitment I can’t go to the village there is somebody going!’ And so you try to understand him out of that burden and bring him back to understand that people must have toilets to reduce diarrhea, to reduce worm infestation, to reduce infections like other infestations you have to get a toilet where to dump that shit." |
| Ability | Healthy populations | “Because the thing is that we work with targets in government and we sign performance contracts, so even if you assign them these duties, the officers will say that it’s not within their performance contracts, so even if they don’t do it, it wouldn’t matter or affect their performance. But when they are taken through this process, they realize that this problem is affecting health issues in communities. They then realize that as an officer, when people are often sick in an area, they won’t be able to mobilize them for any activities, and that he too won’t achieve his targets, so that connection needs to be established.” |
| Ability | Kenya's development | "It’s important that you send reports to Plan Kenya, to the county director and all un sundry it assists in the donors willingness then to avail more funds because Community-led Total Sanitation is a very important activity which needs to be given the seriousness it deserves because people will be sick and the government also spends a lot of money on treatment. For Kenya to develop this is one area where we should be serious and take responsibility" |
| Ability | Kenya's development | "They were interesting because we realized sanitation is also in the constitution of Kenya, sanitation is a right, and is an investment. So it is not the Ministry of Health alone, it is everybody because sanitation affects everybody, it reduces the burden of diseases. The Ministry of Health just participates. So I think from that, that partnership, I think to us as a ministry, it was an achievement." |
| Ability | Ministry specific links | "There is no much difference but the only difference is that when a baby girl goes to school because of the biological make up she has body changes that make her at times miss school and that is the menstruation and so the toilet that we are putting in to term it as gender sensitive is that they have a place to wash or change themselves whenever they need to. It is also sensitive to people who are physically challenged who can’t climb the staircase and so we make rumps." |
| Ability | Ministry specific links | "there is also a security issue if people of OD in the bushes like women when they go into the bush and are raped or even young girls and that is a security aspect." |
| Ability | Ministry specific links | "so that is already an indication that these people are poor, so it has made me think of putting presence of fecal matter openly disposed is an index of poverty in an area" |
| Ability | Ministry specific links | "You know the total sanitation in our areas of work we never talked about it. It came as an awakening call of involving the youth groups on leading the total sanitation in their areas and is at the focal point. The young people are energetic, open and dynamic and can speak to their parents to embracing liking and constructing the toilets and actually using them." |
| Ability | Ministry specific links | "Another example of the way in which we relate with government departments like for fisheries likewise because with fish it is quite abundant in this area and is nutritious and a delicacy in this area but through fish one can get a number of ailments and when we are talking of poor fecal matter disposal through lakes and through the ocean like with fisherman and people defecating in the oceans and so it is through the fisheries department that we have taken them through CLTS issues and they are of assistance in promoting CLTS and discourage OD in the water source areas ." |
| Ability | Ministry specific links | "Yeah! When we went to trigger I noticed that the best people to approach are children or pupils in primary school. These people tell us whatever is happening and so as far as we have involved other sectors like education but we need to go deep into the root of the schools and introduce the CLTS concept more in schools and should be part of the curriculum in schools. So in the future mentorship programs for CLTS an incorporation of the concept in schools curriculum for pupils and using them as agents for change that is sustainable is key...The value it will add is that the child who will be trained on sanitation surely they will pin point the areas where they defecate. And you remember during the triggering sessions it was children who were able to pin point that ‘in this village there is no toilet! In this household there is no toilet!! And so teaching these young children the issue of CLTS they will like cascade the issue to their community and we are possibly going to have more ODF villages in that way." |
| Ability | Ministry specific links | "I have also been thinking about a situation where we can sensitize the head teachers so that they can pass the information to the society faster." |
| Organizational Factors | Supervisor flexibility | “…and we have our mandate … [the minister] she insisted that I need to concentrate on my mandate and not bother with things that do not concern me because you see you may be spraying stray bullets and then at the end of the day we may not achieve anything.” |
| Organizational Factors | Supervisor flexibility | “the challenge always comes in that CLTS is not a core function within my docket and at times when the trainings are organized they tend to clash with my official … my supervisor is sometimes not willing to let go because he wonders that this is not my core function and not in my job description.” |
| Organizational Factors | Supervisor flexibility | "For example in the beginning we did not have the county government backing us up and even now when we have held sensitization meetings with them they have now really bought the idea and they have now given their pledge that they will support our CLTS activities" |
| Organizational Factors | Competing tasks | "No! No! No! I think the negatives are just from our side in like the fewer numbers for this program and not from the Plan side and that is what I’m saying the negatives are on our side because we are fewer in number with numerous competing tasks and so you find that when a supporting agent may force ‘not force but try to come up with an objective at a particular time then you find you are constrained in one way or the other maybe some cause or some condition what two parties agreed on the other party is not keeping up with the pace and then id doesn’t augur well" |
| Organizational Factors | Decentralization | "[Decentralization] has not been handled very nicely! I can say that; that transition has not been handled very smoothly for instance we had some frontline staff here whose contracts here were ending this month. It is not yet clear if that will be handled. They aren’t sure whether they are still in employment or not! You know those kind of uncertainties are not very good for the job and it makes people uncomfortable at work!” |
| Organizational Factors | Decentralization | "Well! Eeh! Yes! Of course with the new dispensation of the constitution we have of the county government. The good thing we have is that the leadership is just within now like if I want to see the PS-permanent secretary ‘it is just a matter of pressing the button or just walking one staircase just ahead! Yes! and in fact even if I want to meet the minister they are just within here and in fact I’m just from a meeting with the COH-Chief officer of health and the executive committee member for health ...So these are the things that are now motivating me now, six months ago the situation was tough because such people were in Nairobi and you know what you had to meet them there in Nairobi. The good thing is that the officers are now just within the county and that is quite motivating to me. It’s all a matter now of just a walking distance!" |
| Organizational Factors | County budget allocation | “monitoring there is one key element and that is terms of facilitation which we have a very big challenge, like you see the weather here at the upper side of [county] is more or less rainy and you see here we only have here motorbike…so saying that we will do it differently, that will call for a lot of things that will make it very different. So I will say we will just do it the normal way we have been doing it but now with superior skills and knowledge which we got out of the training.” |
| Improved programming outcomes | Cross-ministry collaboration | "as such we have just scheduled a meeting for Plan and the staff that is the M&E the program supervisors to come with a way forward to manage the data so that the data that the partners have like the World vision, Amref and Plan and our data they should have people who are directly in touch and can respond to any body at any given time that someone come to ask for the information .That is something totally new that we are doing...Actually if this meeting that I was planning will pick as I suggested I have not given the date but we had planned it; it will leave a system in place. When I say a system in place that means that someone will be designated for that, we have an M&E and actually we want him to be ready to have extra work and that means that I should not be reporting as a program officer directly I should be reporting through the M&E. So we want to make the office aware of each and every partners support and their activities and so when Plan goes and Amref goes and World vision goes the office will still be there and our monitoring will continue." |
| Improved programming outcomes | Cross-ministry collaboration | “By bringing us together, I have been able to interact with more people, and as we interact we have been able to build new partnerships with the colleagues that I interact with. I must appreciate that one thing that I really benefitted last year, is that during the interaction I was able to know the doctor in charge if the District and through that interaction I was able to secure some resources that I used in my exam period…it has given us a forum where we meet and what they are teaching us we are also sharing experiences and we are also learning on how to work with each other.” |
| Improved programming outcomes | Cross-ministry collaboration | "You see the culmination of the action plan is that it made us realize although previously we were seeing sanitation as a health thing and we were only dealing with towns sewerage, sewer network, but now we are advocating or lobbying for people, you know we construct paths, dams, those ones. We were just seeing people in the catchment area; see how we were in our cocoon? We were not giving the whole community that chance but we were just targeting the people in the catchment area and not replicate it in the open so that they will pollute the path. But now we have dealt with the well-being and health of the common diseases in totality; everybody plays a role. So that is the change. We have put them like our part we want them also to assist us to map the water points and also integrate." |
| Improved programming outcomes | Change in organizations' policies | "As a department I have prepared 4 bills which I have already shared with the MCA’s (members of county assembly) and they have already given their inputs and then from there we go to the stakeholders they also give their inputs and also there is the public participation and they also give their comments. After we get the comments from the public participation we take to the county assembly for debate...Of course you see as a county and in the health department you need to come up with structures like the rules and regulations to govern some of these activities otherwise failure to do so you will miss the direction and therefore first and foremost you must come up with bills related to health that will make me work with ease. So those are the issues and as a manager those are my core responsibilities. That is why I could not carry all the workload like I was telling you!! I could not carry on with CLTs for the past 6 or 7 months ago!! That is why I had to give part of my roles to my deputy to undertake because this time I’m accountable to MCA’s and I’m called there and may need to explain certain issues so I had to delegate the CLTs task." |
| Improved programming outcomes | Change in organizations' policies | "Not really the issue is the harmonization of the water boards and so actually it is still there but here at the county we have prepared a water and sanitation bill and if passed there will be harmonization between that Bill at the national and the Bills at the county...In fact there have been very many meetings and deliberations between the national government and the county government on these two bills because actually every county government it may be having its own sanitation and water Bill and there is also the water Bill 2013 and then there is then the water act 2002 and so all of these executive officers for water and public health have been meeting in Nairobi and everyone brings his Bill and then there is the national Bill and then the county bill and all these are in the process of harmonization." |
| Improved programming outcomes | Change in organizations' policies | "Yes we will work in that way! And what we may need is a blessing from the county government that sustainability issues will be supported and that is why we are saying that if the mentorship and lobby team as such could live us with an Act for CLTs and an Act with the community unit. So long as the community unit has an Act and CLTS has an Act. These can empower the communities to do activities with confidence to do what they are doing right now you know there is a fear that because there is a limit. You see now there is the ICCM (Integrated community case management) that is going on and it’s a very important thing going on right now because child mortality has really reduced terribly from 6 to 5 and now is at 1. So this means that if such a system is not empowered within the law all the gains made will go to the reverse." |
| Improved programming outcomes | Resource mobilization outside Plan Kenya | “the biggest plan that will make us have a breakthrough is to have the governor and the deputy governor and the other people will be brought on board in as far as sanitation is concerned…It is important that they are triggered!! You see when you write a proposal to them now and you want them to support a 1 million activity they will be querying maybe what is 1 million going to do and for what purpose” |
| Improved programming outcomes | Resource mobilization outside Plan Kenya | "And I also feel that if the county government is brought on board the way they were willing to be on board so then I still feel that the success will be realized even when Plan pulls out" |
| Improved programming outcomes | CLTS incorporation into existing programs | "For me personally, I had a feeling that the best approach for us now to succeed, without Plan Kenya, then there was a need for us to go through schools. Through schools because in schools all parents are there, almost. All the community members are there. They have children. At times we could be calling, we have education days in schools, we have parents’ meetings in schools. We can use also children to bring their parents to school, we should teach the children also the dangers of OD. To the higher scale than the way they are being taught in class. In that case it can still improve." |
| Improved programming outcomes | Negative indication of outcomes | "It is continuous and we are up-scaling. We go to other villages once we are done, but we never leave them alone because follow up continues until you make sure it stays remain. Once a Community is triggered and the construct latrines and are using them there still need to be constant monitoring that there will be no relapse |
| Improved programming outcomes | Negative indication of outcomes | "Then we have quarterly review meetings which I have planned and have failed 3 times because of other programs. Like now we are here, for the last 2 weeks, we have not gone to the office. We were in [Place], now we are here, Monday I may not be in the office, there is another meeting, Rotavirus is there, statistics review is there, and you know people are closing the financial year. Plan I think is also winding up to start again in July, so those conflicting tasks, something like that. Programs are coming in and out, you are needed to go, and others you delegate. You can’t be everywhere." |
| Knowledge sharing | Detailed example | "having attended that training, we were invited to…was it last month, we trained youths all over in [County name] and I was able to tackle the area of resource mobilization. There were so many youths, around, at least in each Sub County, around 35." |
| Knowledge sharing | Detailed example | "I have shared with them on the importance of dialogue and during planning I tell that we should never leave out the direct and in direct beneficiaries of any project we should at least plan with them for the project success" |
| Knowledge sharing | Create a system | “If you don’t share knowledge it is like it is not there. So when you share knowledge, you ease the work and also you cannot carry everything on your shoulders you need to leave some of the work to others, so you delegate, such that work continues even without you.” |
| Knowledge sharing | Create a system | "One reason is for continuity. For example we hold government offices and sometimes someone may come in or you may be away or you may get another engagement elsewhere and one day after I have delivered [Trainee's sub-county] to be ODF someone may say ‘[Name] has done a good job! Can you come and do it elsewhere maybe at the county level...So I would like when I leave any other person who is coming in finds a system that is working not an individual’s job! |
| Organizational Factors | Decentralization | "The thing that is motivating me in my work as far as the devolved government is concerned you know we are now are now working closer to the beneficiaries and you know as far as the beneficiaries are equally happy that things can go much easily and quicker since they have to move to an area that in nearer and within their reach and that really motivates me. Equally with the devolved government everything is within our reach not only the devolved government but also the people who are now executing the duties are also fully motivated." |
| Organizational Factors | Decentralization | “for example we sat down here somewhere last year, and we identified that same local government for funding on some activities, which to us was going to raise the living standards of the people in the community but…they said that our ministry and therefore our directorate was not devolved and therefore the resources they have is for the devolved government or for the departments which are at national level. So we sat down here, took that proposal to the minister, from the minister’s level, he accepted and he was positive about. But when we went now to the other levels, because he is not the decision maker, he was told that the adult education, and the primary and the secondary are not devolved.” |
| Organizational Factors | Division level staffing | "I think we need to involve more people like for instance for me initially when I was getting trained I thought that I was being trained as a manager and the direction was like we shall just get exposed, then the other staff would be trained who would actually do the work. The challenge I have found is that as a manager now, I have very little time to go for these activities. Actually I have missed a number. Apart from the training, I have missed a number when they go for evaluation; I have never gone actually because whenever they want to go I am also held up. Even the previous training, the last one I attended, I went after one day because I was held up in the office. The last one I could not at all. So those are some of the things that are may be a bit of challenges which I didn’t expect...So as at now, I expect that they would be able to cascade it down to the staff that are actually more in the field than I." |
| Organizational Factors | Division level staffing | "No! No! No! I think the negatives are just from our side in like the fewer numbers for this program and not from the Plan side and that is what I’m saying the negatives are on our side because we are fewer in number with numerous competing tasks and so you find that when a supporting agent may force ‘not force but try to come up with an objective at a particular time then you find you are constrained in one way or the other maybe some cause or some condition what two parties agreed on the other party is not keeping up with the pace and then id doesn’t augur well" |
| Organizational Factors | Division level staffing | “we have all staff trained on CLTS including even staff from other departments and even at the division and even at the lower divisions...We also have a system of the community units which also has systems up to the village level so that gives us a good entry point that we can easily use and we are already utilizing also to reach people on the ground.” |
| Organizational Factors | Relations between organizations | "As I said earlier that is a good way to work because it fosters a good relationship the only challenges will be that Plan has been unifying us from a central place now when we live with this other multi-tasking will it over ride CLTS because if it is one program managed then you stick to one program it is easier to manage it will be a challenge." |
| Organizational Factors | Relations between organizations | “The challenges that it will face, key among them can be selfishness, if in any way because in whichever organization there must be leaders, and leaders in terms of who is supposed to coordinate and if this people like for a very long time people have been seeing sanitation as a ministry of health kind of issue so if they don’t incorporate these other people who are not at the Ministry of Health and they want to go by themselves I don’t see them succeeding. Just because of either monetary interest or monetary gains out of that I don’t see them succeeding, this thing is all inclusive because all of us are targeting the community as a client and when it’s all inclusive that is the strength.” |
| Organizational Factors | Relations between organizations | “My representation there in that forum was making sure that they comply with all the rules of environment… When I brought up the issue of EIA [environmental impact assessment] or screening there was reluctance and actually even people saying that NEMA is going too far and because in conducting an EIA somehow you are going to use funds. ” |
| Organizational Factors | Relationship between organizations | “The other challenge that I foresee is that we do not have an account. We were thinking that after sometime when we start mobilizing resources and write proposals this money is supposed to come into a particular account whose account would it be? We debated about that until even right now as we speak that account is within the ministry of health. So people said ‘if that account is within the Ministry of Health how will we know whether it will be utilized properly?’” |
| Organizational Factors | Conflicting priorities | "if an NGO has a program and it has scheduled activities that you have to be at this level at this particular time and then actually we might have a difficult time because we planned for an activity together but they have again make dates for the same and then communicate back however we have heard a rough time and what I said is that we have to plan specific dates for each and every other activity together not just the NGO coming and saying that they have already planned and so they say they want you to go there ! So with NGOs we have had a difficult time and the other thing is that the clashing periods like whereby we planned for an activity like next week with Plan, Amref or what? And then it reaches a particular time like now we have a national activity like the polio activity that is coming and so you will not prioritize the activity that you had agreed on with the NGO because this is a regional activity and we must plan and fit with the national activities." |
| Environmental, social, and economic factors | National policies | "That is what I talked of they say it is community strategy that is a demand driven process to improve household health from the community but in reality it’s not the case because the policies and roles do not come from the community level they are designed from above. So people are told to adopt the thinking of someone who is seated in a boardroom somewhere remember when you were in [Place] we were told to say our roles and that's what it should be. So for me in the community strategy it should be the person designing his or her own role from the ground." |
