# Supplementary material for: Building capacity for water, sanitation, and hygiene programming: Training evaluation theory applied to CLTS management training in Kenya
Source: Soc Sci Med. 2016 Oct;166:66–76. doi: 10.1016/j.socscimed.2016.08.008 (PMC5034853; doi:10.1016/j.socscimed.2016.08.008)
Supplement: Supplementary file 5 [file mmc5.docx]

**Supplement 5: CLTS success factors and challenges**

**Table S1: Trainee responses regarding CLTS success factors and challenges as recalled by trainees in interview 1, by inclusion in the training manual.** Some factors may have come up during the training in group work that were not in the slides. In these instances, factors given during interviews may be miscategorized as not in training slides.

| **Type of factor mentioned** | **Success** | **Challenge** | **Total** | **In training slides** |
| --- | --- | --- | --- | --- |
| Environmental | 11 | 23 | 34 | Yes |
| Geography | 25 | 5 | 30 | Yes |
| Targeting | 9 | 2 | 11 | Yes |
| Community leadership | 6 | 4 | 10 | Yes |
| Sanitation-health links | 10 | 0 | 10 | Yes |
| CLTS methodology | 3 | 0 | 3 | Yes |
| Government policy and structure | 2 | 1 | 3 | Yes |
| Trainee job responsibilities | 1 | 0 | 1 | Yes |
| Facilitator quality | 5 | 2 | 7 | Yes |
| Human resources | 9 | 3 | 12 | No |
| Socioeconomic | 16 | 15 | 31 | No |
| Cultural | 16 | 14 | 30 | No |
| Community ownership | 17 | 7 | 24 | No |
| Financial resources | 2 | 9 | 11 | No |
|  | | | | |
